# MMP‐9‐dependent proteolysis of the histone H3 N‐terminal tail: a critical epigenetic step in driving oncogenic transcription and colon tumorigenesis

**DOI:** 10.1002/1878-0261.13652

**Published:** 2024-04-10

**Authors:** Yonghwan Shin, Sungmin Kim, Gangning Liang, Woojin An

**Affiliations:** ^1^ Department of Biochemistry and Molecular Medicine, Norris Comprehensive Cancer Center University of Southern California Los Angeles CA USA; ^2^ Department of Urology, Norris Comprehensive Cancer Center University of Southern California Los Angeles CA USA

**Keywords:** colon cancer, histone H3, MMP‐9, proteolysis, transcription

## Abstract

Matrix metalloproteinase 9 (MMP‐9) is a member of the MMP family and has been recently identified as a nuclear protease capable of clipping histone H3 N‐terminal tails (H3NT). This *MMP‐9*‐dependent H3NT proteolysis is critical for establishing an active state of gene transcription during osteoclast differentiation and melanoma development. However, whether H3NT cleavage by *MMP‐9* plays a similar role in other cellular events has not been explored. Here, we dissect the functional contribution of *MMP‐9*‐dependent H3NT clipping to colonic tumorigenesis by using a combination of genome‐wide transcriptome data, ChIP/ChIPac‐qPCR, CRISPR/dCas9 gene‐targeting system, and *in vivo* xenograft models. We show that *MMP‐9* is overexpressed in colon cancer cells and catalyzes H3NT proteolysis to drive transcriptional activation of growth stimulatory genes. Our studies using knockdown and inhibition approaches clearly indicate that *MMP‐9* mediates transcriptional activation and promotes colonic tumorigenesis in a manner dependent on its protease activity toward H3NT. Remarkably, artificial H3NT proteolysis at target gene promoters with dCAS9‐*MMP‐9* is sufficient for establishing their transcriptional competence in colon cancer cells, underscoring the importance of *MMP‐9*‐dependent H3NT proteolysis *per se* in the transactivation process. Our data establish new functions and mechanisms for *MMP‐9* in driving the oncogenic transcription program in colon cancer through H3NT proteolysis, and demonstrate how this epigenetic pathway can be exploited as a potential therapeutic target for cancer treatment.

## Graphical abstract

MMP‐9 is recruited to growth stimulatory genes and functions to mediate H3NT proteolysis at promoter regions. This epigenetic H3NT clipping in turn creates a local chromatin environment favorable to gene expression. MMP‐9 knockdown and inhibition induce a rapid downregulation of target genes and diminish the growth potential of colon cancer cells.

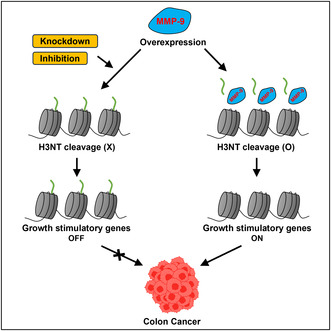

**Abbreviations**
C: coding region
ChIP: chromatin immunoprecipitation
ChIPac‐qPCR: ChIP of acetylated chromatin‐quantitative polymerase chain reaction
H3NT: histone H3N‐terminal tail
MMP‐9: Matrix metalloproteinase 9
MMPs: Matrix metalloproteinases
P: promoter region
PCA: principal component analysis
RNA‐seq: RNA sequencing
RT‐qPCR: real‐time‐quantitative polymerase chain reaction
TFs: transcription factors

## 1 Introduction

Colon cancer is a leading cause of cancer deaths worldwide and is widely acknowledged to arise from genetic alterations in genes encoding important regulators of cell growth and proliferation [1, 2, 3]. However, accumulating evidence suggests that epigenetic aberrations also contribute to the development of colon cancer through mis‐regulation of gene expression and perturbation of normal cell growth states [4, 5, 6, 7]. Since these alterations lead to the initiation, promotion, and progression of colon cancer, manipulating epigenetic factors to correct abnormal transcription programs has a great therapeutic potential for colon cancer. In support of this notion, it has been shown that aberrant DNA methylation and histone modification contribute to the process of colon carcinogenesis by inducing inadvertent silencing of tumor suppressor genes as well as unintended activation of potential proto‐oncogenes [8, 9, 10, 11, 12, 13, 14, 15]. Our previous work also showed that *VprBP*, a recently identified kinase, phosphorylates histone H2A, epigenetically inactivates tumor suppressor genes, and drives colonic tumorigenesis [16, 17]. Considered together, these findings provide a clear indication that the targeted DNA and histone modifications have a direct function in establishing distinct epigenetic states in a group of genes and initiating a cascade of transcription events that underlie the development and progression of colon cancer. However, it still remains elusive whether other epigenetic changes also drive oncogenic transcription program and increase the risk of developing colon cancer.

Matrix metalloproteinases (MMPs) represent a family of zinc‐dependent endopeptidases with a highly conserved sequence and similar domain structure [18, 19, 20, 21, 22, 23, 24]. To date, 23 distinct proteinases have been identified as members of the MMP family in human cells and have shown to cleave a wide variety of extracellular matrix substrates, including collagen, gelatin, fibronectin, laminin, and proteoglycan [25, 26]. *MMP‐9* is a member of the MMP family and exerts regulatory control over the cell matrix composition through its zinc‐dependent proteolytic activity [27, 28]. Analogous to other MMP family members, *MMP‐9* is initially synthesized as a 92‐kDa proenzyme in an enzymatically inactive state and subsequently processed to a fully active 82‐kDa form by extracellular proteases originating from the endoplasmic reticulum [29]. Although classically viewed as a protease to alter extracellular environments, *MMP‐9* is more recently recognized as a possible factor regulating a range of nuclear processes [28, 30, 31]. In accordance with this idea, our studies unveiled nuclear localization and transactivating function of *MMP‐9* during osteoclatogenesis and melanomagenesis [27, 32, 33, 34]. Through continued investigation into *MMP‐9* functionality, we discovered that *MMP‐9* catalyzes the proteolytic cleavage of the histone H3 N‐terminal tail (H3NT) protruding from nucleosomes with K18 as the P1 site [27, 32, 34]. This *MMP‐9*‐dependent H3NT proteolysis constitutes a crucial process for driving pro‐osteoclastogenic and pro‐melanomagenic transcription programs, thereby promoting osteoclast differentiation and melanoma development. These findings establish a new mechanism of action of *MMP‐9* involving its nuclear localization and H3NT‐targeted activity. We also developed a technique called ChIP of acetylated chromatin (ChIPac) to identify H3NT‐cleaved regions and demonstrated a strong correlation between H3NT proteolysis and target gene transactivation [32]. Of special relevance to the current report, previous studies demonstrated that *MMP‐9*‐induced ECM remodeling and membrane protein cleavage play an important role in the development, invasion, and metastasis of colon cancer [35, 36, 37]. In addition to these observations, more recent investigations also implicated *MMP‐9* in regulating distinct nuclear pathways in colon cancer cells, stressing the importance of nuclear *MMP‐9* function in the process of colonic tumorigenesis [38, 39]. Despite these advances, however, little is known about nuclear reactions that *MMP‐9* controls and the underlying molecular mechanisms involved in its oncogenic properties.

Here, we report that *MMP‐9* is overexpressed and proteolytically cleaves H3NTs in the nuclei of colon cancer cells. Functional studies revealed that *MMP‐9*‐dependent H3NT proteolysis is necessary for the expression of growth stimulatory genes in colon cancer cells. Knockdown and inhibition of *MMP‐9* impairs H3NT clipping, inactivates a large set of growth stimulatory genes, and diminishes oncogenic cell growth. We also demonstrate that dCAS9‐*MMP‐9* fusion can catalyze H3NT proteolysis and activate transcription when artificially positioned in target gene promoters. Moreover, similarly blocking H3NT‐targeted *MMP‐9* protease activity in xenograft models effectively attenuates the expression of target genes and impedes the growth of colonic tumor xenografts.

## 2 Materials and methods

### 2.1 Cell lines, constructs, and antibodies

All cell lines (NCM460, Caco2, HCT15, HCT116, HT29, LOVO, RKO, and SW620) were purchased from ATCC (American Type Culture Collection, Manassas, VA, USA). NCM460 (RRID:CVCL_0460) and LOVO (RRID:CVCL_0399) cells were cultured in RPMI1640 medium supplemented with 10% fetal bovine serum (FBS). Caco2 (RRID:CVCL_0025), HCT15 (RRID:CVCL_0292), HCT116 (RRID:CVCL_0291), HT29 (RRID:CVCL_A8EZ), LOVO, RKO (RRID:CVCL_0504), and SW620 (RRID:CVCL_0574) cells were cultured in Dulbecco's modified Eagle's medium with 10% FBS. Cells were authenticated by STR profiling and tested for mycoplasma contamination. For the mammalian expression of MMP‐9, the MMP‐9 cDNA was amplified through PCR and inserted into the lentiviral expression vector pLenti‐Hygro (Addgene, Watertown, MA, USA) containing a 5′ 3 × FLAG coding sequence. The MMP‐9 E402A catalytic dead mutant expression vector was generated by site‐directed mutagenesis using the Q5 Site‐Directed Mutagenesis Kit (New England Biolabs, Ipswich, MA, USA). Antibodies used in this study are as follows: anti‐H2A, anti‐H2B, anti‐H3, anti‐H4, and anti‐MMP‐9 antibodies from Abcam (Cambridge, MA, USA); anti‐Actin and anti‐HA antibodies from Proteintech (Rosemont, IL, USA); and anti‐Lamin B1 antibody from Thermo Fisher Scientific (Waltham, MA, USA).

### 2.2 Cell lysate and chromatin preparation and western blot analysis

Whole cell lysates and cytoplasmic and nuclear fractions were obtained from NCM460, Caco2, HCT15, HCT116, HT29, LOVO, RKO, and SW620 cells using M‐PER™ Mammalian Protein Extraction Reagent (Thermo Scientific, Waltham, MA, USA). Chromatin was extracted from the cultured cells following the protocol described in our previous studies [27, 32, 33, 34]. Western blotting was conducted with the prepared samples as described previously [27, 32, 33, 34].

### 2.3 Co‐immunoprecipitation

SW620 colon cancer cells were washed with cold PBS and lysed in cell lysis buffer (50 mm Tris–HCl, pH 7.4, 150 mm NaCl, 1 mm EDTA, 1% Triton X‐100, 1 mm PMSF, and 1× Roche protease inhibitor cocktail). Whole cell lysates were incubated with MMP‐9 antibody for overnight followed by incubation with Protein A/G agarose beads (Santa Cruz Biotechnology, Santacruz, CA, USA). Beads were washed four times with lysis buffer, and the binding of endogenous MMP‐9 and H3 was analyzed by western blot.

### 2.4 Tissue samples

10 colon tumor samples along with 10 normal tissue counterparts were obtained (January–April 2021) from Dr. Heinz‐Josef Lenz at the Division of Medical Oncology, Norris Comprehensive Cancer Center at the University of Southern California, USA [16, 17]. Using fresh frozen specimens, we conducted western blot analyses to examine the expression levels of H3 and MMP‐9. Approval for this study was granted by the University of Southern California's IRB Ethics Committee (Protocol HS‐06000678), and all patients provided written informed consent as per the committee's regulations. The study methodologies were conformed to the standards set by the Declaration of Helsinki.

### 2.5 Mass spectrometry

Human H3 containing a C‐terminal FLAG tag was expressed in SW620 cells for a duration of 48 h. Whole cell lysates were prepared as described [32, 34], and ectopic intact and NT‐cleaved forms of H3 protein were purified using immunoprecipitation with anti‐FLAG M2 antibody (Sigma, St. Louis, MO, USA) in a precipitation buffer (20 mm Tris–HCl, pH 7.3, 300 mm KCl, 0.2 mm EDTA, 20% glycerol, and 0.1% Nonidet P‐40). The purified H3 proteins were separated using 15% SDS/PAGE and stained with Coomassie blue. Fast‐migrating bands corresponding to H3 were excised from the gels, subjected to trypsin digestion, and analyzed by liquid chromatography–tandem mass spectrometry (LC–MS/MS) following the procedure described in our recent studies [32, 34].

### 2.6 RNA interference

The DNA oligonucleotides (5′‐CATTCAGGGAGACGCCCATTT‐3′) designed to target the coding region of MMP‐9 were annealed and ligated into the lentiviral expression vector pLKO.1 (Addgene, Watertown, CA, USA). Lentiviral particles were generated in 293T cells by transfecting plasmids encoding VSV‐G, NL‐BH, and the MMP‐9‐specific shRNA. Two days post‐transfection, the viral supernatants were collected and utilized to infect SW620 cells in the presence of polybrene (8 μg·mL^−1^). The infected SW620 cells were selected for 2 weeks with puromycin (2 μg·mL^−1^) to establish MMP‐9‐depleted cell lines. For rescue experiments, MMP‐9‐depleted cells were subsequently infected with lentiviruses expressing shRNA‐resistant MMP‐9 wild type or catalytic dead mutant E402A and selected for 2 weeks in the presence of hygromycin (300 μg·mL^−1^).

### 2.7 RNA‐seq

RNA was extracted from SW620 cells using the Qiagen RNeasy kit (Qiagen Inc., Valencia, CA, USA), and its quality was assessed using the Agilent Bioanalyzer with the DNA1000 kit according to the manufacturer's instructions. After generating strand‐specific libraries with the KAPA Stranded mRNA‐Seq Kit and KAPA mRNA Capture Beads (Kapa Biosystems Inc., Wilmington, MA, USA), they were pooled, denatured, and diluted to 15 pM prior to clonal clustering on the sequencing flow cell using the Illumina cBOT Cluster Generation Station and Cluster Kit v3‐cBot‐HS. Sequencing was performed on the clustered flow cell using 1 × 50 single‐end reads on the Illumina HiSeq as in our recent studies [34, 40]. Base conversion of the sequenced data was carried out using OLB version 1.9, and the resulting sequences were demultiplexed and converted to Fastq format using CASAVA version 1.8 (Illumina, San Diego, CA, USA) [32, 34]. Subsequently, the RNA‐seq reads were aligned to the hg38 GENCODE version 29 reference genome using STAR 2.6.1d [41, 42]. The aligned reads were then quantified at the gene level, and gene counts were normalized using the upper quartile normalization method. Principal component analysis was performed using the normalized gene counts, and differentially expressed genes were identified using the Gene Specific Algorithm from Partek Flow software (Partek Inc., Chesterfield, MO, USA) [40]. A volcano plot was generated based on the fold change and false discovery rate of genes, with a false discovery rate cutoff of 5 × 10^−4^ and an absolute fold change greater than 2, enabling the statistical identification of significantly differentially expressed genes. Gene ontology analysis of the differentially expressed genes was conducted using the Ingenuity Pathway Analysis tool (IPA version 52 912 811) (Qiagen Inc., Germantown, MD, USA). Heatmaps were generated by calculating the Z score of gene expression levels using the heatmap.3 function from the Generalized Minimum Distance r package [43].

### 2.8 RT‐qPCR

Total RNA was extracted from colon cancer cells using the RNeasy kit (Qiagen, Hilden, Germany) and converted to cDNA using the iScript cDNA Synthesis Kit (Bio‐Rad, Hercules, CA, USA). RT‐qPCR was conducted using the one‐step QuantiTect SYBR Green Real‐time PCR kit (Qiagen, Hilden) according to the manufacturer's instructions. The primer sequences employed for RT‐qPCR are provided in Table S1. All reactions were run in triplicate, and results were averaged.

### 2.9 ChIPac and ChIP assays

ChIPac assays were performed using chromatin that underwent fixation with 10 μm methylene blue and acetylation with 20 mm acetic anhydride following the established protocol [27, 32, 33, 34]. The crosslinked chromatin was subjected to sonication and immunoprecipitation using immobilized H3K14ac and H3CT antibodies on protein A/G‐PLUS agarose. ChIP assays were conducted using the H3K14ac and H3CT antibodies and the ChIP assay kit (Millipore, Burlington, MA, USA) as recently described [27, 32, 33, 34]. DNA fragments were recovered from precipitated protein–DNA complexes using Qiagen kit after reversal of crosslinking at 65 °C and subjected to qPCR reactions with primers specific to the promoter (P) and coding regions (C) of FGF2, LY6L and TRIM46 genes. The primer sequences used for ChIPac/ChIP experiments are provided in Table S2. The specificity of amplification was verified through melting curve analysis, and all reactions were run in triplicate.

### 2.10 Cell viability and colony formation assays

Control and MMP‐9‐depleted/rescued SW620 cells were seeded in 96‐well plates at a density of 2 × 10^3^ cells per well for cell viability assessment over a 5‐day period using the MTT assay kit (Sigma). To evaluate the effects of MMP‐9 inhibition, MTT assays were also conducted after treating SW620 cells with the MMP‐9 inhibitor MMP9‐I (10 nm) for 5 consecutive days. For colony formation assays, control and MMP‐9‐depleted SW620 cells were seeded at a density of 1 × 10^3^ cells per well in 6‐well plates and allowed to form colonies over a 2‐week period. SW620 cells were also treated with DMSO or the MMP‐9 inhibitor MMP9‐I for 72 h prior to colony formation assays. The colonies in each well were stained with 0.5% crystal violet, photographed, and counted using ImageJ software (ver. 1.53 k, U. S. National Institutes of Health, Bethesda, MD, USA) [40]. All assays were performed in triplicate, and the presented results represent the average of three independent experiments.

### 2.11 CRISPR/dCas9‐based H3NT cleavage assays

The dCas9‐MMP‐9 expression vector was constructed by fusing human MMP‐9 to the catalytically inactive nuclease codon‐optimized S. pyogenes Cas9 (dCas9) cDNA in an expression vector using by the CAG promoter as in our recent study [40]. The pPlatTET‐gRNA2 vector (Addgene 82559, Cambridge, MA, USA) was digested and purified using a Qiagen gel extraction kit (Qiagen Inc., Valencia). MMP‐9 wild type (wt) or MMP‐9 E402A catalytic dead mutant were cloned from complementary DNA (cDNA) using specific primers. The digested and purified pPlatTET‐gRNA2 vector was then ligated with the MMP‐9 wt or MMP‐9 E402A fragments using T4 DNA ligase (Thermo Scientific). To generate specific guide RNAs (sgRNAs), gene datasets for FGF2, LY6L, and TRIM46 were selected from UCSC and used to design 58‐bp oligos containing the respective sgRNA sequences using the CHOPCHOP tool (https://chopchop.cbu.uib.no/) [40]. The designed oligos were synthesized and used for PCR amplification with primers (Table S3). The amplified fragments were purified and subjected to a recombination reaction using the In‐Fusion HD Cloning Plus Kit protocol (Takara Bio, San Jose, CA, USA) with the digested pPlatTET‐MMP‐9 wt/E402A construct. MMP‐9‐depleted SW620 cells were transfected with plasmids encoding dCas9, dCas9‐MMP‐9 wt, or dCas9‐MMP‐9 E402A using the FuGENE® HD Transfection Reagent (Promega, Madison, WI, USA). 48 h post‐transfection, cells were passaged, and 50 μg·mL^−1^ Neomycin was added 3 h after plating. Media was exchanged 48 h post‐transfection, and cells were passaged every other day starting from 4 days after the initial replating. Neomycin selection was maintained for a total of 7 days. To study the enzymatic activity, transactivate effects, and growth regulatory activities of dCas9‐MMP‐9 fusions in transfected cells, western blotting, RT‐qPCR, cell viability, and colony formation assays were performed.

### 2.12 Mice xenograft

*In vivo* experiments were conducted using 8‐week‐old athymic male nude mice [(Crl:NU(NCr))‐Foxn1nu] (Charles River, Wilmington, MA, USA) housed in specific pathogen‐free conditions. To investigate the knockdown effects, the mice were randomly divided into two groups (*n* = 6 mice per group), and control or *MMP‐9*‐depleted SW620 cells (1 × 10^7^ cells) were subcutaneously injected into the right flank of each mouse. Body weights were monitored every 3 days for a total of 24 days. Tumor volumes were estimated by measuring the width (W) and length (L) of the tumors using a digital caliper and calculated using the formula: TV = W2L/2. For inhibitor treatment, SW620 colon cancer xenografts were established for 5 days, and randomly divided into two groups. The mice in each group were then treated with either DMSO or *MMP‐9* inhibitor *MMP9*‐I (50 μg·kg^−1^) every 3 days for a period of 24 days. At the conclusion of the experiment, the mice were euthanized, and the tumors were excised for further analysis as described previously [34]. All animal operations including breeding, welfare, and execution were conducted with the approval and guidance of the Ethical Animal Care and Institutional Animal Care and Use Committee of University of Southern California (Approval No. 20722‐AM004).

### 2.13 Statistical analysis

All quantitative data are reported as mean ± standard deviation (SD). Statistical analyses of datasets were conducted using Student's two‐tailed *t*‐test or two‐way ANOVA followed by Bonferroni post‐hoc test, utilizing graphpad prism software (GraphPad Software Inc., San Diego, CA, USA) for all experimental analyses. A *P*‐value < 0.05 was considered statistically significant.

## 3 Results

### 3.1 *MMP‐9* overexpression and H3NT proteolysis are prevalent in colon cancer cells

**Fig. 1:**
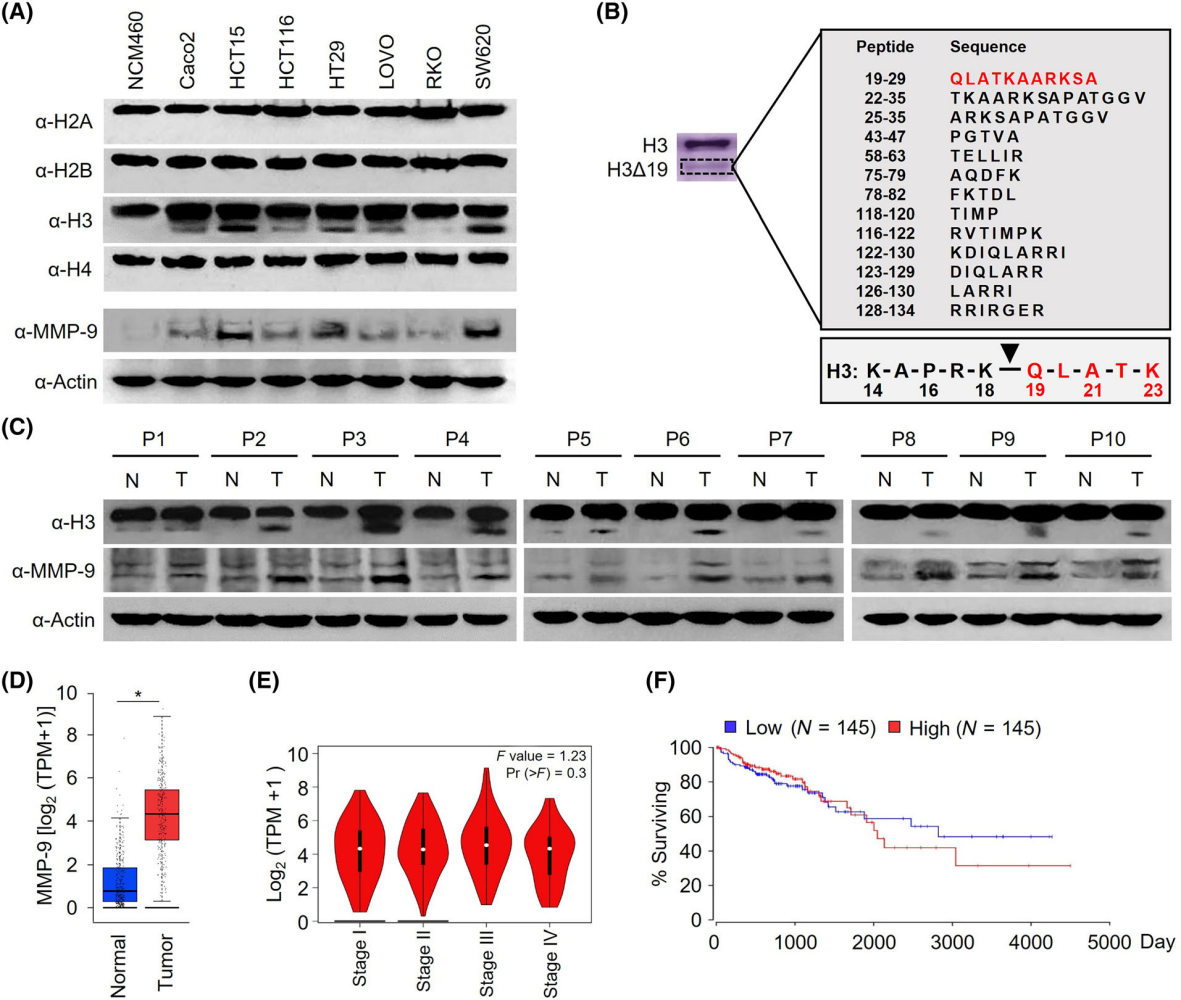
MMP‐9 is highly expressed and H3NT is proteolytically cleaved in colon cancer cells. (A) Whole cell lysates and chromatin fractions were prepared from colon cancer (Caco2, HCT15, HCT116, HT29, LOVO, RKO, and SW620) and normal colon (NCM460) cells and subjected to western blotting with antibodies against H2A, H2B, H3, H4, and MMP‐9. Actin was used as loading control. Shown are the representative results of three independent immunoblot experiments. (B) Peptide sequences identified by liquid chromatography tandem mass spectrometry (LC–MS/MS) analysis of the fast‐migrating H3 band excised from purified H3 protein fractions. Arrowhead indicates the cleavage site. (C) Whole cell lysates and chromatin fractions were prepared from 10 human colon tumor and their adjacent normal tissue samples. The relative levels of H3 and MMP‐9 in the purified samples were determined by western blot analysis. Actin was used as a loading control. N, normal tissue; T, tumor tissue. Shown are the representative results of three independent immunoblot experiments. (D) Box and whisker plots show the altered expression of MMP‐9 in colon adenocarcinoma (COAD) in TCGA database. The numbers of normal (275, blue) and tumor (349, red) samples are analyzed. Boxes represent 25–75% of values, and black lines in boxes represent median expression values. *P* values were calculated using one‐way ANOVA. *P < 0.01. (E) Violin plots display the expression patterns of MMP‐9 in different clinical stages of colon adenocarcinoma (COAD) using the Gene Expression Profiling Interactive Analysis (GEPIA) online analysis tool (http://gepia.cancer‐pku.cn/). (F) The Kaplan–Meier (KM) curve represents overall survival rate based on MMP‐9 expression status in 145 colon cancer patients. These patients were analyzed using the oncolnc online tool (http://www.oncolnc.org/).

Given that cancer cells are often characterized by epigenetic alterations, it was reasonable to expect that aberrant composition and modification of histone proteins might be a critical mechanism leading to transcriptional dysregulation and colonic carcinogenesis. As a first step toward exploring this possibility, we isolated nuclei from seven colon cancer cell lines (Caco2, HCT15, HCT116, HT‐29, LOVO, RKO, and SW620) and one normal colon cell line (NCM460) and extracted chromatin fractions. Interestingly, our western blot analysis revealed the presence of a faster‐migrating H3 band in chromatin fractions prepared from the majority of colon cancer cell lines (Fig. 1A). In contrast, no fast‐migrating band was observed in chromatin fractions extracted from the normal cell line NCM460 (Fig. 1A), indicative of a possible involvement of proteolytic H3 cleavage in the development of colon cancer. Since we used an antibody recognizing the C‐terminus of H3 for our analyses, these results also reflect the proteolytic clipping of H3 occurring specifically within NT region. To determine how many amino acids of H3NT region are proteolytically removed in colon cancer cells, the C‐terminally FLAG‐tagged H3 was expressed in SW620 cells and affinity purified with M2‐agarose beads under stringent conditions (300 mm KCl, 0.1% Nonidet P‐40). After SDS/PAGE and Coomassie blue staining of the purified H3 protein fractions, the fast‐migrating H3 band was subjected to liquid chromatography–tandem mass spectrometry (LC–MS/MS) analysis (Fig. 1B). This analysis failed to detect H3 peptide fragments preceding Q19, thus establishing K18 as the P1 residue of H3 cleavage site in colon cancer cells.

We have recently reported that *MMP‐9* is highly expressed and clips off H3NT with K18 being the P1 site during osteoclastogenesis and melanomagenesis [27, 32, 34]. Therefore, it was reasonable to speculate that *MMP‐9* may also be of particular importance for H3NT proteolysis reactions in colon cancer cells. Adding support to this idea, our western blotting and RT‐qPCR detected *MMP‐9* expression at much higher levels in colon cancer cells compared to normal colon cells (Fig. 1A, Fig. S1A). Also, cellular levels of *MMP‐9* are very well correlated with extents of H3NT proteolysis in colon cancer cells in most cases (Fig. 1A). In view of H3NT proteolysis occurring in the nucleus, we also prepared nuclear and cytoplasmic fractions from the cell lines and analyzed the subcellular localization patterns of *MMP‐9* by western blotting. In agreement with our previous observation, *MMP‐9* was detected in similar levels in both the nuclear and cytoplasmic fractions (Fig. S2). As another approach to examine the relationship between *MMP‐9* and H3NT proteolysis in colon cancer cells, we repeated western blotting with colonic tumor and adjacent normal tissue samples from 10 patients. These experiments demonstrated that MMP‐9 expression is significantly elevated in nine out of 10 colonic tumor samples and is positively correlated with the extent of H3NT proteolysis (Fig. 1C, Fig. 1B,C). Consistent with these findings, our analysis of colon cancer datasets available from the Cancer Genome Atlas (TCGA) showed that MMP‐9 has significantly higher expression levels in tumor samples compared to normal samples (Fig. 1D). To further explore the association between elevated *MMP‐9* expression and patient outcomes in colon cancer, we conducted an analysis of *MMP‐9* expression levels alongside survival curves. In checking *MMP‐9* expression levels in four stages of colon cancer, *MMP‐9* overexpression was detected in the four stages of colon cancer with only minor variations (Fig. 1E). Kaplan–Meier survival analysis and log‐rank test comparison also revealed somewhat uneven distribution of cases between the generated survival curves (Fig. 1F). Together, these initial observations link *MMP‐9* to H3NT proteolysis in colon cancer cells and provide a rationale for our continued investigation in more direct fashion.

### 3.2 *MMP‐9* catalyzes H3NT cleavage in colon cancer cells

**Fig. 2:**
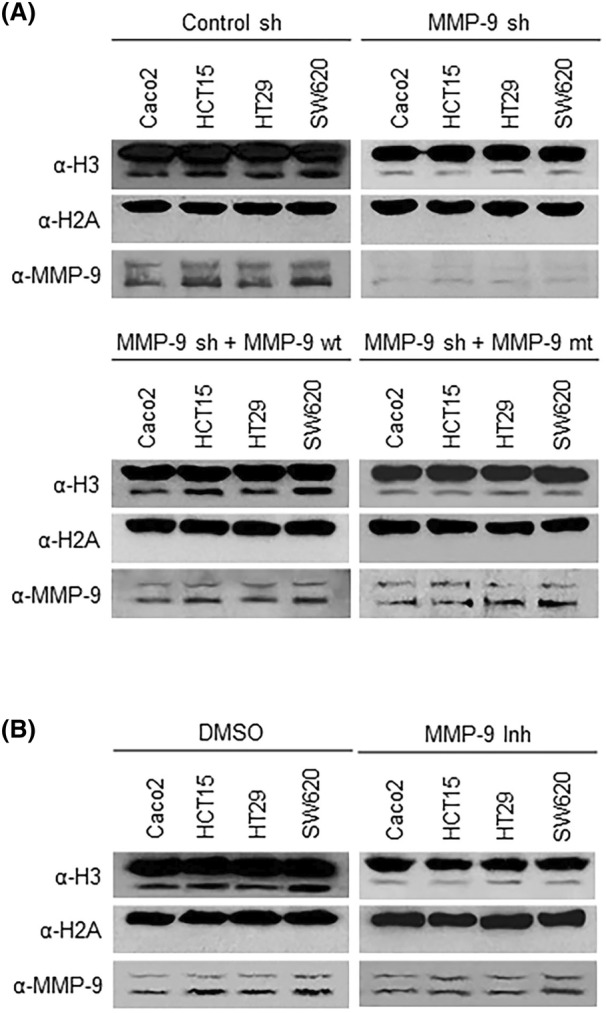
MMP‐9 is the protease responsible for H3NT proteolysis in colon cancer cells. (A) Four colon cancer cell lines (Caco2, HCT15, HT29, and SW620), which exhibited the highest levels of H3NT clipping in Fig. 1A, were infected with lentiviral MMP‐9 shRNA. To rescue the knockdown effects, MMP‐9‐depleted cells were transfected with MMP‐9 wild type or E402A catalytic dead mutant. Nuclear lysates and chromatin samples were then prepared and subjected to western blotting with antibodies against H3, H2A, and MMP‐9. Shown are the representative results of three independent immunoblot experiments. (B) Western blot analysis was conducted as in (A), but with cells treated with MMP‐9 inhibitor MMP9‐I. Shown are the representative results of three independent immunoblot experiments.

Given the established role for *MMP‐9* in catalyzing H3NT proteolysis in osteoclast and melanoma cells, the positive correlation between H3NT cleavage and *MMP‐9* expression levels in colon cancer cells suggests a potential involvement of *MMP‐9* in H3NT clipping process. To explore this possibility, Caco2, HCT15, HT29, and SW620 cells were depleted of *MMP‐9* using a lentiviral shRNA infection system and then transfected with *MMP‐9* wild type or E402A catalytic dead mutant. We first confirmed the successful depletion of endogenous *MMP‐9* following stable transfection with *MMP‐9* shRNA and comparable expression of ectopic *MMP‐9* wild type/mutant in the four depleted cell lines (Fig. 2A; note that the upper and lower bands shown in *MMP‐9* western blotting represent 92 kDa latent and 84 kDa active forms, respectively.). Chromatin fractions were then prepared from cell lysates as in Fig. 1A, and western blot analysis with the C‐terminal histone H3 antibody was employed to detect both intact and NT‐cleaved forms of H3 proteins in the fractions. As expected, our analysis detected high levels of H3NT proteolysis and *MMP‐9* expression in mock‐depleted control cells (Fig. 2A). Contrarily, we observed much lower levels of H3NT proteolysis upon stable knockdown of *MMP‐9*. In checking the rescue potential of ectopic *MMP‐9*, we found that the expression of *MMP‐9* wild type in *MMP‐9*‐depleted cells restored H3NT clipping to the original level, whereas *MMP‐9* E402A catalytic dead mutant was unable to restore the level of H3NT proteolysis (Fig. 2A). Since *MMP‐9* inhibitor *MMP9*‐I is effective in blocking *MMP‐9* protease activity [27, 32, 34], we also evaluated its effects on H3NT proteolysis in colon cancer cells as an extension of the knockdown studies. The results of these efforts showed that treatment with *MMP‐9* inhibitor *MMP9*‐I for 5 days significantly impairs the process of H3NT proteolysis in colon cancer cells (Fig. 2B). Adding support to these observations and in accordance with the concept of *MMP‐9*‐H3 interaction as an important process for *MMP‐9*‐dependent H3NT proteolysis, our immunoprecipitation of endogenous *MMP‐9* also showed a stable association of histone H3 in colon cancer cells (Fig. S3). All these results strongly imply that *MMP‐9* is the protease capable of recognizing H3 and catalyzing H3NT clipping in colon cancer cells.

### 3.3 *MMP‐9* knockdown and inhibition attenuate the growth potential of colon cancer cells

**Fig. 3:**
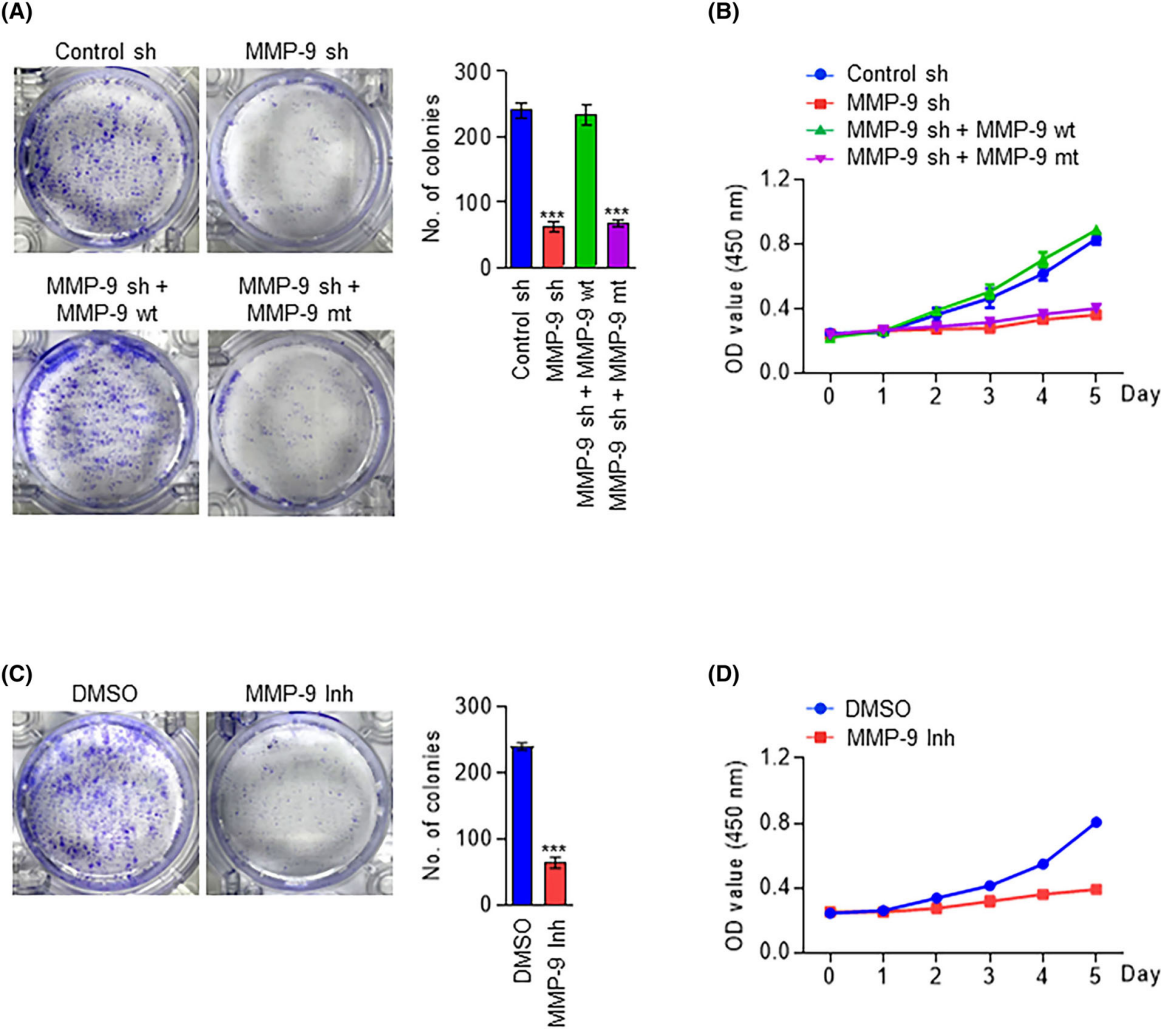
MMP‐9 protease activity is essential for the growth of colon cancer cells. (A, B) MMP‐9‐depleted SW620 cells were complemented with MMP‐9 wild type or E402A catalytic dead mutant, and their growth was assessed by colony formation assays after 14 days of culture (A) and MTT assays over a 5‐day period (B). The results are presented as the mean ± SD of three experiments conducted in triplicate. *P* values were calculated using paired *t*‐tests. ***P < 0.001 versus control sh. (C, D) SW620 cells were treated with MMP‐9 inhibitor MMP9‐I, and their growth was measured by colony formation assays and MTT as in (A and B). Data represent the mean ± SD of three independent experiments conducted in triplicate. *P* values were calculated using paired *t*‐tests.***P < 0.001 versus DMSO.

Although the above‐described studies demonstrated a role for *MMP‐9* in catalyzing H3NT proteolysis in colon cancer cells, it is not clear whether H3NT clipping by *MMP‐9* is of any importance for the growth and viability of colon cancer cells. For the purpose of addressing this question, we performed MTT assays over a period of 5 days, daily monitoring changes in cell growth rates in response to *MMP‐9* knockdown. As summarized in Fig. 3B, an apparent decrease in cell growth rate was observed upon stable depletion of *MMP‐9* in SW620 colon cancer cells. To examine whether *MMP‐9*‐dependent removal of H3NT is critical for the observed growth‐stimulatory effects, we also conducted rescue experiments. These assays revealed that the expression of *MMP‐9* wild type restored the growth capacity of *MMP‐9*‐depleted SW620 cells (Fig. 3B). On the other hand, *MMP‐9* catalytic dead mutant failed to recover the original growth rate of *MMP‐9*‐depleted SW620 cells (Fig. 3B), thus suggesting that H3NT clipping activity of *MMP‐9* is necessary for *MMP‐9* function in promoting the growth of colon cancer cells. In an effort to gain support for the MTT assay results, we also evaluated the impact of *MMP‐9* knockdown on the clonogenic potential of colon cancer cells over a period of 14 days. The results from these experiments indicate that *MMP‐9* depletion causes about 70% decrease in the ability of SW620 colon cancer cells to grow and expand into a colonal population (Fig. 3A). Also, when we checked the rescue potential of ectopic *MMP‐9*, the expression of *MMP‐9* wild type, but not *MMP‐9* catalytic dead mutant, in *MMP‐9*‐depleted cells fully restored the colony forming capacity of the cells (Fig. 3A). The involvement of *MMP‐9*‐dependent H3NT proteolysis in promoting the growth capacity of colon cancer cells was also confirmed by similar colony formation and MTT assays showing a substantial decrease in the growth rate of SW620 cells after treatment with *MMP‐9* inhibitor *MMP9*‐I (Fig. 3C,D). Together, these data argue strongly for the direct action of *MMP‐9* on colon cancer and support the concept that *MMP‐9* exerts its oncogenic function via H3NT proteolysis.

### 3.4 *MMP‐9*‐dependent H3NT proteolysis drives transcriptional dysregulation in colon cancer cells

**Fig. 4:**
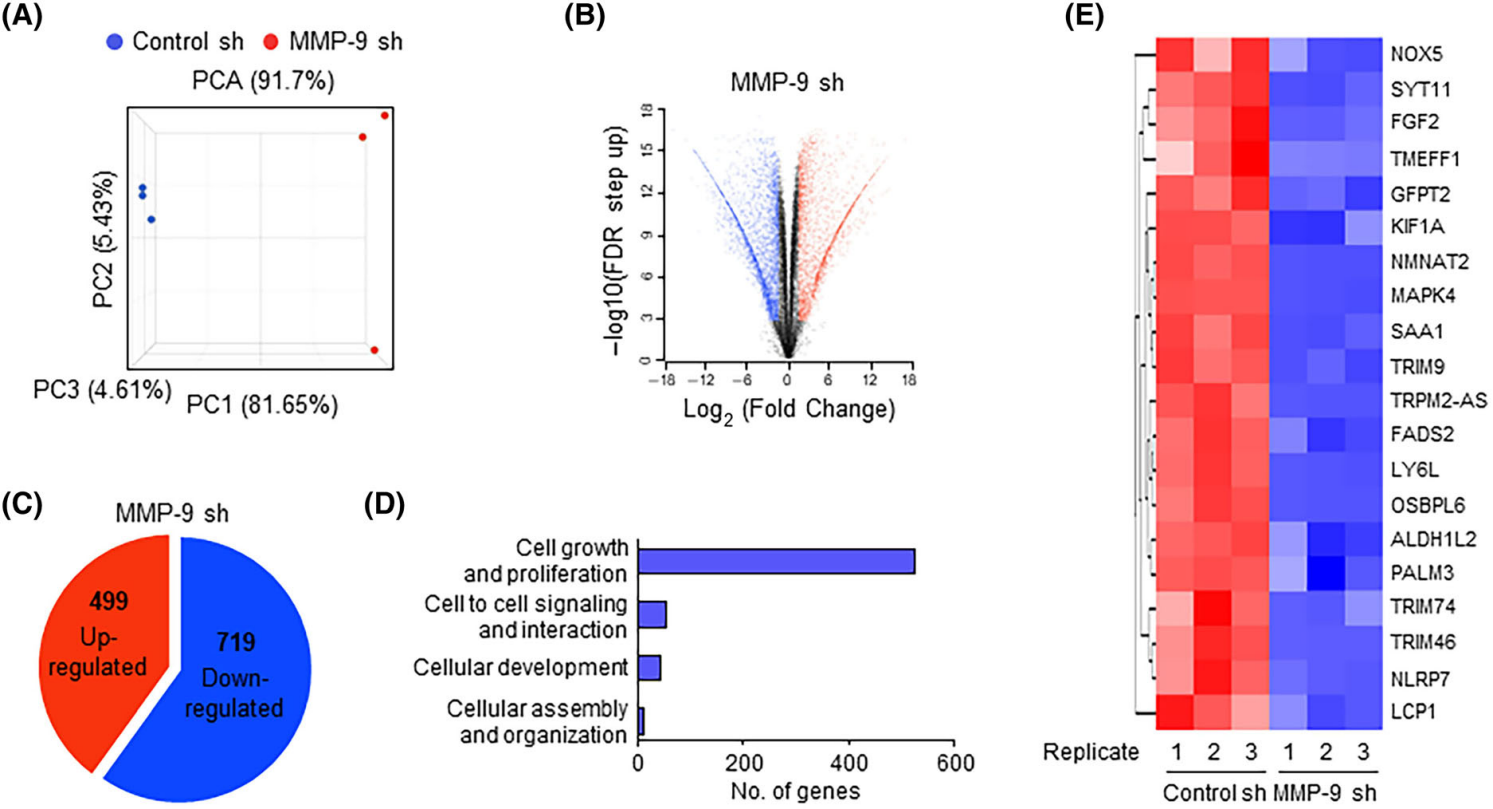
MMP‐9 transactivates growth‐stimulatory genes in colon cancer cells. (A) Principal Component Analysis (PCA) was performed on RNA‐seq datasets obtained from SW620 colon cancer cells. The MMP‐9 knockdown (MMP‐9 sh) group is shown in red, and the control (control sh) group is represented in in blue. Each group comprised three replicates. (B) A volcano plot illustrates the RNA‐seq datasets with −log10 (FDR step up) displayed on the *Y*‐axis and the fold change of gene expression between the MMP‐9 knockdown and control groups on the *X*‐axis. Genes exhibiting modulation following MMP‐9 depletion are highlighted in blue (downregulated) and red (upregulated). Data show three biological replicates. (C) Venn diagram displays genes that were significantly upregulated or downregulated (> 2 fold; FDR < 5 × 10^−4^) in MMP‐9‐depleted SW620 cells compared to mock‐depleted control cells. (D) Gene ontology analysis was conducted using the Ingenuity Pathway Analysis (IPA version 52912811) tool developed by Qiagen to examine the activated genes after MMP‐9 knockdown. *P*‐value < 0.005, FDR < 0.005. (E) Heatmap presents the expression levels (*Z*‐scores) of the top 20 genes encoding positive regulators of cell growth and proliferation which are inactivated upon MMP‐9 depletion. High and low expression levels are shown in red and blue, respectively. Data show three biological replicates.

**Fig. 5:**
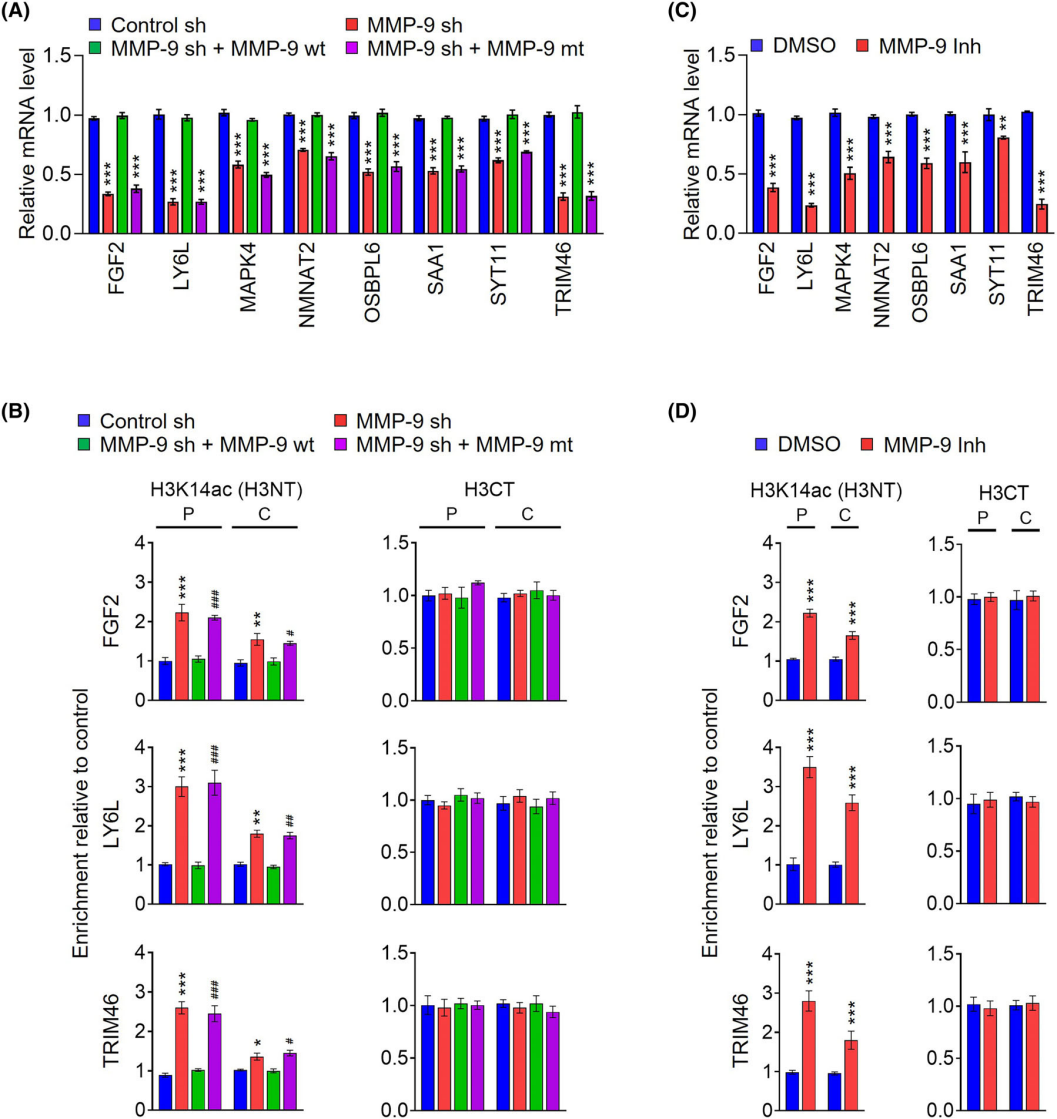
MMP‐9‐dependent H3NT proteolysis is critical for the active state of target genes. (A) SW620 cells were infected with lentiviral nontargeting shRNA (control sh) or MMP‐9 shRNA (MMP‐9 sh), and RNA samples were isolated and subjected to RT‐qPCR analysis using primers listed in Table S1. To recover knockdown effects, MMP‐9‐depleted SW620 cells transfected with MMP‐9 wild type (MMP‐9 sh + MMP‐9 wt) or E402A catalytic dead mutant (MMP‐9 sh + MMP‐9 mt) and analyzed. All transcription levels were normalized to GAPDH. Data are presented as the mean ± SD (*N* = 3); *P* values were determined using paired *t*‐tests. ***P < 0.001 versus control sh. (B) ChIP assays were performed in groups described in a using H3K14ac and H3CT antibodies. The ChIP DNAs were analyzed by real‐time PCR with primer pairs amplifying the promoters (P) and coding regions (C) of the FGF2 (upper panel), LY6L (middle panel), and TRIM46 (lower panel) genes. The primers used are listed in Table S1. Data are represented as the mean ± SD obtained from independent triplicate experiments. *P < 0.05, **P < 0.01, and ***P < 0.001 versus control sh; ^#^ *P* < 0.05, ^##^ *P* < 0.01, and ^###^ *P* < 0.001 versus MMP‐9 sh + MMP‐9 wt. (C) SW620 cells were treated with MMP‐9 inhibitor MMP9‐I for 5 days, and the expression of MMP‐9 target genes was analyzed by RT‐qPCR as in (A). Data were expressed as mean ± SD (*N* = 3); *P* values were calculated using paired *t*‐tests. **P < 0.01, and ***P < 0.001 versus DMSO. (D) ChIP assays were conducted as described in (B), but using MMP‐9 inhibitor MMP9‐I‐treated SW620 cells. Data are represented as the mean ± SD of independent triplicate experiments. *P* values were calculated using paired *t*‐tests. ***P < 0.001 versus DMSO.

Since *MMP‐9* functions as a transcription regulator by cleaving H3NT in osteoclast and melanoma cells, we next wanted to examine whether *MMP‐9*‐dependent H3NT proteolysis described above plays any role in inducing oncogenic transcription program. Toward this end, we performed RNA sequencing (RNA‐seq) with total RNA isolated from control and *MMP‐9*‐depleted SW620 colon cancer cells. Our initial evaluation of RNA‐seq data using principal component analysis (PCA) confirmed a clear separation among samples for each group, while close clustering of replicates from groups indicated minimal variability in the quality of analyzed replicates (Fig. 4A). Using a two‐fold cutoff, we identified a total of 1218 genes differentially expressed upon stable depletion of *MMP‐9* in SW620 cells from RNA‐seq‐based transcriptome profiling (Fig. 4B,C, Fig. S4). Among those genes, 719 genes were repressed, and 499 genes were activated in response to *MMP‐9* knockdown (Fig. 4C). Consistent with our previous reports implicating *MMP‐9*‐dependent H3NT clipping in oncogenic transcription events, gene ontology analysis of 719 upregulated targets also identified cell growth and proliferation as the most highly ranked category (Fig. 4D). The role for *MMP‐9* in colon cancer was further supported by the fact that our analysis of the leading‐edge subset in the gene set detected 20 genes encoding positive regulators of cell growth and proliferation (Fig. 4E). As an experiment to validate our RNA‐seq data, we conducted reverse transcription quantitative PCR (RT‐qPCR) analysis of 8 target genes that are highly ranked in our analysis and encode factors stimulating cell growth in several types of cancers including colon cancer. As summarized in Fig. 5A, our analysis showed that *MMP‐9* knockdown caused 40–70% decreases in their mRNA levels. The ectopic expression of *MMP‐9* wild type in *MMP‐9*‐depelted cells allowed them to return to the original transcription levels (Fig. 5A). Importantly, when *MMP‐9* E402A catalytic dead mutant was expressed in *MMP‐9*‐depleted cells, target genes were still expressed at very low levels, underscoring the importance of *MMP‐9*‐dependent H3NT proteolysis for target gene activation in colon cancer cells (Fig. 5A). Similar RT‐qPCR assays after treatment with *MMP‐9* inhibitor *MMP9*‐I also detected the inactive state of target genes in SW620 cells, results of the incapability of *MMP‐9* to induce H3NT cleavage and thus target gene potentiation (Fig. 5C).

To investigate whether the observed function of *MMP‐9* in activating target gene transcription reflects its direct effects on target genes, we next checked the levels of H3NT proteolysis at target genes by using our recently developed technique called ChIP of acetylated chromatin (ChIPac) assay. In this assay, chromatin is crosslinked with methylene blue, fragmented by sonication, and treated with acetic anhydride to acetylate all lysine residues. Then, chromatin fragments containing intact H3NT are specifically precipitated by H3K14ac antibody, and a reduced PCR signal relative to the control ChIPac reactions using an H3CT antibody is indicative of H3NT proteolysis. In the present study, the precipitated DNA was amplified by quantitative PCR (qPCR) with two primer sets specific for the promoters (P) and coding regions (C) of target genes. We decided to focus our analysis on three target genes *FGF2*, *LY6L*, and *TRIM46* encoding cellular components that stimulate cell growth, division, and proliferation. Our initial ChIPac assays detected low levels of H3K14ac in the promoter and coding regions of three representative target genes in mock‐depleted control SW620 cells (Fig. 5B). However, H3K14ac signals were much higher, especially at promoter regions, when ChIPac experiments were performed in *MMP‐9*‐depleted cells (Fig. 5B), a finding in line with the idea that *MMP‐9* targets the process of initiating transcription for its transactive action through H3NT proteolysis. These results were validated by rescue experiments demonstrating that ectopic expression of *MMP‐9* in *MMP‐9*‐depleted cells restored H3NT clipping to levels quantitatively similar to those observed with mock‐depleted control cells. It was also apparent in our parallel ChIPac assays that ectopic expression of *MMP‐9* E402A catalytic dead mutant failed to override H3NT proteolysis defects caused by *MMP‐9* knockdown (Fig. 5B). These observations were further corroborated by additional ChIPac experiments in which exposure of SW620 colon cancer cells to *MMP‐9* inhibitor *MMP9*‐I still allowed *MMP‐9* occupancy but almost completely crippled H3NT proteolysis at target genes (Fig. 5D). We thus concluded that *MMP‐9*‐dependent H3NT proteolysis is directly linked to the process of driving the expression of growth‐stimulatory genes in colon cancer cells.

### 3.5 dCAS9‐*MMP* *‐9* artificially cleaves H3NTs and transactivates *MMP‐9* targets

**Fig. 6:**
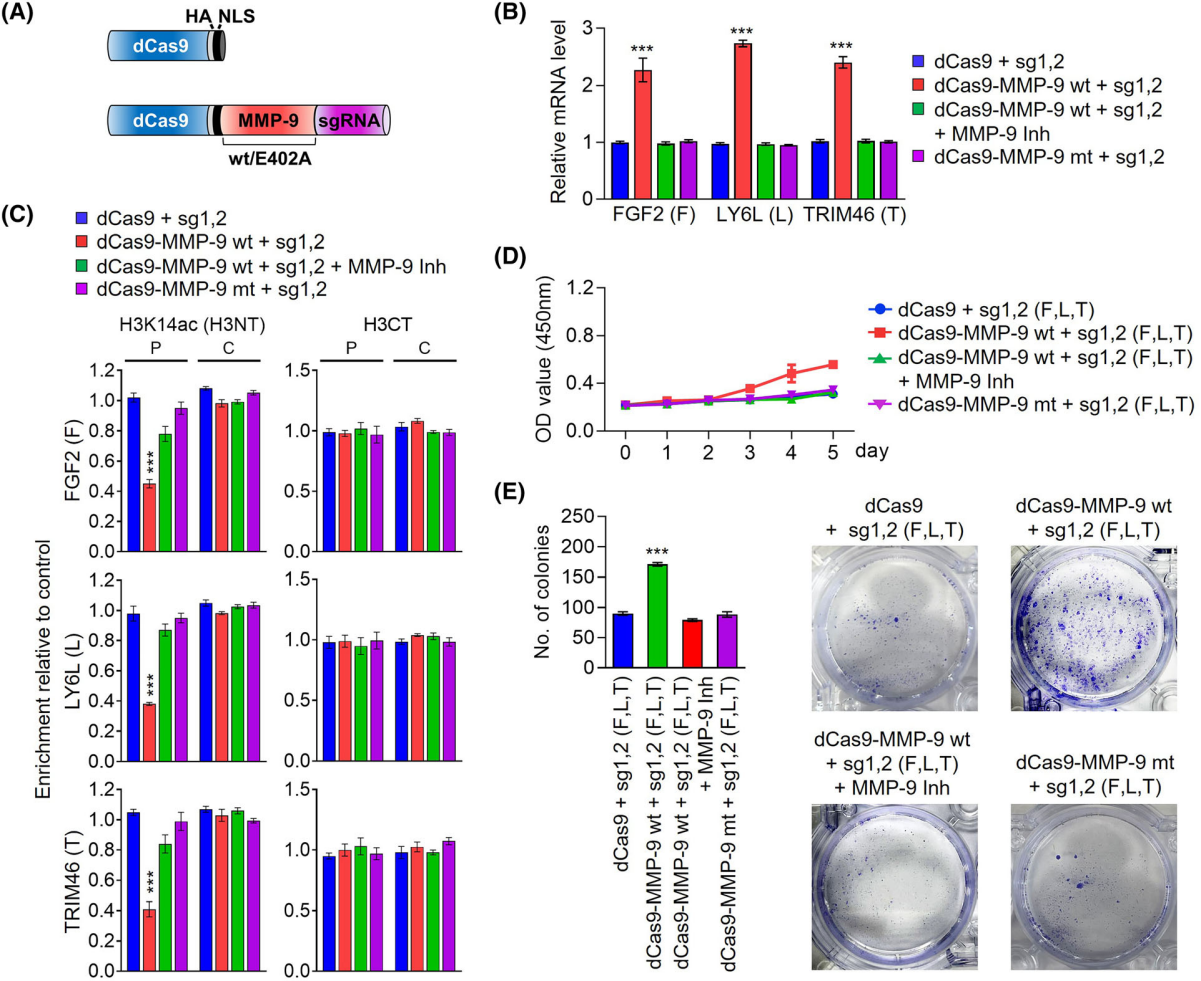
Promoter‐bound dCas9‐MMP‐9 activates target gene transcription. (A) Schematic representation of the CAG‐based constructs driving the expression of dCas9 or dCas9‐MMP‐9 wt/E402A catalytic dead mutant, along with sgRNAs targeting FGF2, LY6L and TRIM46 genes. (B) MMP‐9‐depleted SW620 cells were transfected with the indicated constructs expressing dCas9 or dCas9‐MMP‐9 and sgRNA1, 2 for 48 h. Total RNA was isolated and analyzed by RT‐qPCR using specific primers for the FGF2, LY6L, and TRIM46 genes. Data represents as the mean ± SD (*N* = 3); *P* values were calculated using paired *t*‐tests. ***P < 0.001 versus control. (C) dCas9/dCas9‐MMP‐9 and sgRNA1, 2 were expressed in MMP‐9‐depleted SW620 cells as in B. ChIP‐qPCR analysis was performed to assess the levels of H3K14ac at the promoters (P) and coding regions (C) of the FGF2, LY6L and TRIM46 genes. Data are represented as the mean ± SD obtained from independent triplicate experiments. *P* values were calculated using paired *t*‐tests. ***P < 0.001. (D) dCas9‐MMP‐9 was guided to target genes as outlined in C, and changes in cell growth were monitored using MTT assays over a period of 5 days. The results represent the mean ± SD of three experiments performed in triplicate. (E) Colony formation assays were conducted in SW620 cells after selective upregulation of FGF2, LY6L and TRIM46 genes using CRISPR/dCas9 system as described in (B). Data represents the mean ± SD of three independent experiments in triplicate wells; *P* values were calculated using paired *t*‐tests. ***P < 0.001.

Along with our demonstration of the causal role that *MMP‐9*‐dependent H3NT proteolysis plays in activating a cluster of growth‐stimulatory gene in colon cancer cells, our ChIPac‐qPCR analysis indicated that H3NT proteolysis in the promoter region of target genes correlates with *MMP‐9* transactivation. Although these data strongly implicate MMP‐9‐dependent H3NT proteolysis in transcription process, it is still not clear whether H3NT proteolysis *per se* is necessary and sufficient for *MMP‐9* action in driving target gene activation. If *MMP‐9* induces target gene transcription by catalyzing H3NT proteolysis, it appeared plausible that artificially tethering *MMP‐9* to target genes is sufficient to re‐establish an active state of transcription in *MMP‐9*‐depleted colon cancer cells. We thus decided to employ CRISPR/dCas9‐based system for directing *MMP‐9*‐dependent H3NT proteolysis to target genes and exploring its ability to reactivate target genes in *MMP‐9*‐depleted SW620 cells. Accordingly, we constructed a series of pPlatTET‐gRNA2 all‐in‐one vectors expressing dCas9‐*MMP‐9* wild type (wt) or catalytic dead mutant (E402A) and single guide RNAs (sgRNAs) recognizing the promoters or proximal coding regions of the three target genes (*FGF2*, *LY6L*, and *TRIM46*) (Fig. 6A). In measuring the impact of these dCas9‐*MMP‐9* fusions, we found that the expression of dCas9‐*MMP‐9* wild type together with promoter‐binding sgRNA 1 or 2 in *MMP‐9*‐depleted SW620 cells transcriptionally potentiates *FGF2*, *LY6L*, and *TRIM46* genes (Fig. S5). Also, by using sgRNA 1 and 2 together, we were able to generate a more pronounced activation of *FGF2*, *LY6L*, and *TRIM46* genes in these assays (Fig. 6B, Fig. S5). However, coding region‐binding sgRNA 3 and 4 minimally altered the levels of target gene transcription in dCas9‐*MMP‐9* wild type‐transfected cells (Fig. S5), thus again implying that transcription initiation is the step mainly regulated by *MMP‐9*‐dependent H3NT proteolysis. Consistent with expectations from these results, simultaneously targeting dCas9‐*MMP‐9* to promoter and coding regions by sgRNA 1, 2, 3 and 4 together activated target genes at levels comparable with those observed with sgRNA 1 and 2 pair (Fig. S5). Under identical assay conditions, *MMP‐9* inhibitor *MMP9*‐I treatment significantly impaired the transactivation potential of dCas9‐*MMP‐9* wild type at target genes, and dCas9‐*MMP‐9* E402A catalytic dead mutant failed to generate any detectable activation of target gene transcription (Fig. 6B, Fig. S5).

Based on the transcription results described above, we next tested the ability of dCas9‐*MMP‐9* to catalyze H3NT proteolysis at target genes by ChIPac‐PCR. Co‐transfection of dCas9‐*MMP‐9* wild type with sgRNA 1 and 2 pair in *MMP‐9*‐depleted SW620 cells led to high levels of H3NT clipping in the promoter regions of *FGF2*, *LY6L*, and *TRIM46* genes (Fig. 6C). In monitoring the extent of H3NT proteolysis in MMP‐9‐depleted cells expressing dCas9‐*MMP‐9* wild type together with sgRNA 3 and 4 pair, we observed H3NT clipping only in the coding regions (Fig. S6A). When all four sgRNAs were used together with dCas9‐*MMP‐9* wild type in our assays, H3NT proteolysis was also repeatedly detected in both the promoter and coding regions of target genes, despite clipping levels being lower in coding regions (Fig. S6B). However, in agreement with transcription data, all the sgRNAs failed to trigger H3NT proteolysis at the target genes in dCas9‐*MMP‐9* E402A catalytic dead mutant‐expressing cells as well as *MMP‐9* inhibitor *MMP9*‐I‐treated cells (Fig. S6B). Given that *FGF2*, *LY6L*, and *TRIM46* genes encode the components of growth control system, we also wished to examine whether delivering dCas9‐*MMP‐9* with sgRNA 1 and 2 pair to their promoter regions can lead to changes in cell growth rate. In our MTT and colony formation assays, an increase in cell growth rates and colony numbers was evident, when dCas9‐*MMP‐9* wild type was co‐expressed with sgRNA 1 and 2 pair in *MMP‐9*‐depleted SW620 cells (Fig. 6D,E). Conversely, when sgRNA 1 and 2 were replaced by sgRNA 3 and 4 and when dCas9‐*MMP‐9* E402A mutant was used instead of dCAS‐*MMP‐9* wild type, we failed to observe any augmentation of growth and colony formation potential of *MMP‐9*‐depleted SW620 cells in our assays (Fig. S7A,B). Also, sgRNA 1, 2, 3 and 4 together stimulated the growth and colony formation of *MMP‐9*‐depleted SW620 cells expressing dCas9‐*MMP‐9* wild type, similar to that observed with sgRNA 1 and 2 pair (Fig. S7C,D). These data together with ChIPac‐qPCR results above argue persuasively that *MMP‐9* drives oncogenic transcription program in an H3NT clipping‐independent manner, and reinforce the conclusion that *MMP‐9* can accurately establish H3NT proteolysis‐induced gene activation and growth‐stimulatory effects if stably recruited to the target genes.

### 3.6 *MMP‐9* knockdown and inhibition suppress colonic tumorigenesis

**Fig. 7:**
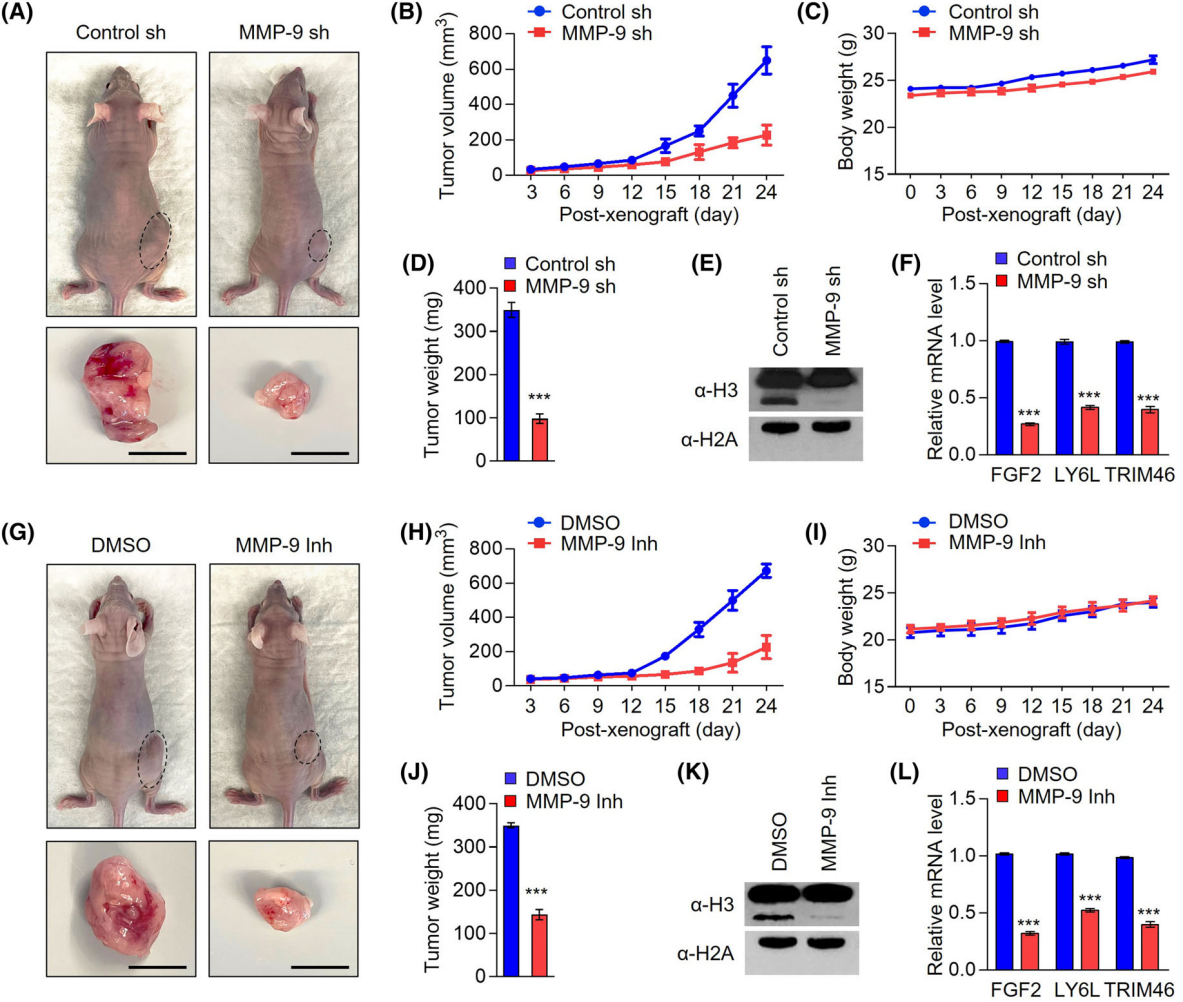
MMP‐9 knockdown and inhibition suppress colon tumor growth *in vivo*. (A) Colon cancer xenografts were generated by subcutaneous injection of mock‐depleted control (control sh) and MMP‐9‐depleted (MMP‐9 sh) SW620 cells into the right side of mouse skin. After 24 days, mice were sacrificed, and SW620 colon cancer xenografts were surgically excised and photographed (lower panel, scale: 1 cm) (*n* = 6 biologically independent experiment). (B) Volume of colon cancer xenograft tumors was measured every 3 days following SW620 cell injection into mice. Data are represented as the mean ± SD (*n* = 6). (C) Body weights of mice carrying colon cancer xenografts were measured every 3 days after injection of mock‐depleted control or MMP‐9‐depleted SW620 cells. Data are represented as the mean ± SD (*n* = 6). (D) SW620 colon cancer xenografts were excised as in (A), and their weights were measured and expressed in milligrams. The data are presented as the mean ± SD (*n* = 6); *P* values were calculated using paired *t*‐tests. ***P < 0.001 versus control sh. (E) Western blot analysis was performed on the excised SW620 colon cancer xenografts using H3 and H2A antibodies. Data are representative of three independent experiments. (F) Relative mRNA levels of FGF2, LY6L and TRIM46 genes in SW620 colon cancer xenografts obtained 24 days post‐injection were determined by RT‐qPCR. Data represent the mean ± SD of independent triplicate experiments; *P* values were calculated using paired *t*‐tests. ***P < 0.001 versus control sh. (G) SW620 colon cancer xenografts were established as in a and treated with DMSO or MMP‐9 inhibitor MMP9‐I for 24 days. On day 24 after treatment, mice were sacrificed, and SW620 colon cancer xenografts were surgically excised and photographed (lower panel, scale: 1 cm), (*n* = 6 biologically independent experiment). (H) The volume of SW620 colon cancer xenograft tumors was measured every 3 days over a 24‐day MMP‐9 inhibitor MMP9‐I treatment period. Data are represented as the mean ± SD (*n* = 6). (I) Body weights of mice carrying colon cancer xenografts were measured every 3 days after injection of DMSO or MMP‐9 inhibitor MMP9‐I. Data are represented as the mean ± SD (*n* = 6). (J) After treating with MMP‐9 inhibitor MMP9‐I for 24 days, colon cancer xenografts were excised from mice, and tumor weight was measured. Data represent the mean ± SD (*N* = 6); ***P < 0.001 versus DMSO. *P* values were calculated using paired *t*‐tests. Data are represented as the mean ± SD (*n* = 6). (K) Western blot analysis was performed on the excised SW620 colon cancer xenografts using H3 and H2A antibodies. Data are representative of three independent experiments. (L) Relative mRNA levels of FGF2, LY6L and TRIM46 genes were measured by RT‐qPCR. Data represent the mean ± SD (*N* = 6) of independent triplicate experiments; *P* values were calculated using paired *t*‐tests. ***P < 0.001 versus DMSO.

As an extension of the above‐described *in vitro* studies that established a stimulatory function for *MMP‐9* on the growth of colon cancer cell lines, it was also important to investigate the contribution of H3NT‐targeted *MMP‐9* protease activity to colonic tumorigenesis *in vivo*. For this objective, we generated xenograft models by subcutaneously injecting mock‐depleted control or *MMP‐9*‐depleted SW620 colon cancer cells into the right hind legs of nude mice. In tracking the growth of these SW620 xenograft tumors at 3‐day intervals for a period of 24 days, a significant reduction of SW620 xenograft growth was observed after the stable knockdown of *MMP‐9* (Fig. 7A,B,D). Given the demonstrated reliance of *MMP‐9* function on H3NT clipping activity, it was also reasonable to expect similar effects of *MMP‐9* inhibitor *MMP9*‐I in our assays. In this regard, we observed that the proliferative capacity of SW620 xenografts was also reduced by an average of 60% following the administration of 50 μg·kg^−1^ *MMP‐9* inhibitor *MMP9*‐I every 3 days over a period of 24 days (Fig. 7G,H,J). Another key question arising from these xenograft growth data is to what extent *MMP‐9* knockdown and inhibition affect H3NT proteolysis in SW620 colon cancer xenograft models. To address this question, we sacrificed mice, harvested colonic SW620 xenograft tumors, and prepared xenograft lysates. In accordance with our *in vitro* study, our western blot analysis showed that *MMP‐9* knockdown and inhibition almost completely abolished H3NT proteolysis in SW620 xenograft tumors (Fig. 7E,K). As a mechanistic basis for observed repressive effects, our RT‐qPCR data also showed that target genes were significantly suppressed shRNA‐mediated knockdown and *MMP‐9* inhibitor *MMP9*‐I‐induced inhibition of *MMP‐9* in SW620 xenograft tumors (Fig. 7F,L). Importantly, *MMP‐9* knockdown and inhibition are well tolerated with no body weight loss observed in the SW620 xenograft model (Fig. 7C,I), suggesting that they may be useful for effective colon cancer therapy. These *in vivo* observations, together with those described earlier, indicate that *MMP‐9* plays a crucial role in driving colonic tumorigenesis likely through H3NT proteolysis and provide a conceptual frame work for future studies using preclinical models.

## 4 Discussion

Several proteases such as Cathepsin L and *JMJD5/7* have been reported to cleave H3NT in different cell types and described as influencing chromatin dynamics and functional properties, thereby playing a crucial role in the epigenetic regulation of gene expression [44, 45, 46]. It is anticipated that more proteases responsible for clipping H3NT in specific nuclear processes continue to be identified and characterized as a critical epigenetic regulator. *MMP‐9*, just like other MMPs, has been widely investigated as a protease responsible for the degradation of extracellular matrix components [23, 24, 31]. However, our recent studies with much greater attention to its nuclear function revealed that *MMP‐9* can cleave H3NT to establish active transcription states during osteoclast differentiation and melanoma development [27, 32, 33, 34]. Our genome‐wide and cellular characterization of MMP‐9 indicates that *MMP‐9* overexpression and consequent H3NT clipping upregulate a group of genes critical for osteoclastogenesis and melanomagenesis [27, 32, 33, 34]. Despite the epigenetic role for *MMP‐9*‐dependent H3NT proteolysis established in these earlier investigations, its possible involvement in other pathological processes is still not clear. In this report, we demonstrate that the H3NT clipping activity of *MMP‐9* is directly linked to altered transcription program during the development of colon cancer.

In our initial characterization of H3NT clipping process in colon cancer cells, *MMP‐9* was identified as an enzyme that mediates proteolytic cleavage of H3NT and induces abnormal cell growth. Consistent with our previous reports [34], we also found that the 18‐amino acid N‐terminal tail domain of H3 is removed by *MMP‐9* in colon cancer cells, suggesting a conserved specificity of *MMP‐9* among different cell types. To search for *MMP‐9*‐regulated genes in colon cancer cells, we analyzed the genome‐wide regulatory potential of MMP‐9 by RNA‐seq of colon cancer cells. Expectedly, there were significant changes in the expression of growth stimulatory genes in response to *MMP‐9* knockdown, and the altered genes were especially enriched with functions that contribute to cell growth and cancer development. Among most significantly altered genes, we selected *FGF2*, *LY6L*, and *TRIM46* for further study because their gene products are known to increase the viability and malignant proliferation of cancer cells. In probing their promoter and proximal coding regions by ChIPac, we detected much higher levels of *MMP‐9* occupancy and H3NT proteolysis at the promoter region compared to the coding region. With the use of knockdown and inhibition approaches, we also confirmed a major role of *MMP‐9*‐dependent H3NT proteolysis in activating growth‐stimulatory genes as well as promoting colon tumor growth *in vivo*. These findings are in good agreement with those of our previous reports demonstrating that *MMP‐9* mainly localizes to target gene promoters and generates locus‐specific H3NT proteolysis to regulate their transcriptional competence in melanoma cells [47, 48]. Our current work also provides strong support for the epigenetic action of *MMP‐9* dependent of H3NT proteolysis in executing oncogenic transcription programs necessary for colonic tumorigenesis and represents a significant extension of its biological function as a specific H3NT protease (Fig. S8). In fact, the findings described in this study provide the first demonstration that histone tail proteolysis directly contributes to colonic tumorigenesis through mis‐regulation of gene transcription.

Another significant feature of the present study is the use of CRISPR‐dCAS9 targeting system to confirm H3NT clipping‐dependent action of *MMP‐9* that establish and maintain an active transcription state of growth‐stimulatory genes in colon cancer cells. In this study, targeting dCAS9‐*MMP‐9* to a promoter region triggered H3NT proteolysis and resulted in reactivation of target genes in *MMP‐9*‐depleted colon cancer cells. On the other hand, H3NT proteolysis by dCAS9‐*MMP‐9* in the coding region was not sufficient for the establishment of active chromatin environment at target genes. This difference in efficiency between targeting promoter and coding regions suggests that *MMP‐9*‐dependent H3NT proteolysis induces relief of nucleosome‐mediated repression at the promoters to facilitate the formation of preinitiation complex which is critical for the initiating step of transcription. The inability of dCAS9‐*MMP‐9* catalytic dead mutant to enhance transcription in *MMP‐9*‐depleted cells indicates that H3NT proteolysis is prerequisite for *MMP‐9* action during transcription process. The functional importance of *MMP‐9*‐dependent H3NT proteolysis is further supported by the observation that target genes cannot be activated by dCAS9‐*MMP‐9* in cells treated with *MMP‐9* inhibitor *MMP9*‐I. Although more work is required to clearly understand the molecular details, the results presented in this targeting study are sufficient to demonstrate the significance of *MMP‐9*‐dependent H3NT proteolysis in driving oncogenic gene expression in colon cancer cells. Moreover, simultaneous activation of three target genes was effective in stimulating growth capacity of colon cancer cells in our assays. This finding suggests that *MMP‐9*‐dependent H3NT clipping induces aberrant activation of multiple target genes, which act synergistically to convert normal cells to cancer cells under the pathological conditions. In fact, our data represent the first direct indication that a specific transcription process is dependent on *MMP‐9*‐dependent H3NT proteolysis *per se*. Application of dCAS9‐*MMP‐9* along with different sgRNAs to target specific *MMP‐9* responsive genes in our future study will undoubtedly facilitate our efforts to delineate whether H3NT proteolysis is also required for *MMP‐9*‐triggered transactivation of other responsive genes in colon cancer cells.

Based on the molecular characteristics and functional properties that we have established for *MMP‐9*‐dependent H3NT clipping, we propose the following model for *MMP‐9* transactivation in the process of colonic tumorigenesis. After translocating from the cytoplasm to the nucleus, nuclear *MMP‐9* is brought to target genes by interaction with specific transcription factors (TFs) at the initial stage of gene transcription. After its stable localization at target genes, *MMP‐9* proteolytically cleaves H3NTs, mostly over promoter nucleosomes. This H3NT truncation affects histone‐DNA interactions and substantially decreases nucleosome stability, thereby establishing and maintaining an active state of nucleosomes at target gene promoters. This model implies that *MMP‐9* nuclear localization and recruitment could be the rate‐limiting steps for the gene‐specific action of *MMP‐9* in colon cancer cells. This model also has implications for H3NT‐dependent gene silencing, because un‐cleaved intact H3NTs will stabilize both inter and intra‐nucleosomal interactions to establish inactive transcription state. An important question remaining to be answered is about the initial recruitment mechanism of *MMP‐9* to target genes for its H3NT proteolytic and transactivation functions in colon cancer cells. One possibility is that gene‐specific transcription factors directly interact with *MMP‐9* and constitute a major mechanism for recruiting *MMP‐9* and triggering transcriptional activation at target loci. Adding support to this idea, we recently purified factors that can interact with *MMP‐9* in SW620 colon cancer cells and detected a stable association of several sequence‐specific DNA‐binding factors (Not shown). Therefore, functional characterization of *MMP‐9*‐interacting factors identified from different types of cancer cells will be important to understand cancer type specificity and target gene selectivity of *MMP‐9* in the process of establishing distinct transcription programs and unravel a layer of new regulatory mechanisms governing *MMP‐9*‐dependent H3NT proteolysis in cancer cells.

## 5 Conclusions

In this report, we demonstrate that *MMP‐9* is overexpressed and responsible for catalyzing H3NT proteolysis in colon cancer cells. Our genome‐wide transcriptome analysis shows that growth‐regulatory genes are selectively targeted and activated by *MMP‐9*‐dependent H3NT proteolysis in colon cancer cells. *MMP‐9* establishes the active state of those target genes in an H3NT clipping‐dependent manner, because *MMP‐9* wild type, but not *MMP‐9* catalytic dead mutant, largely overrides the effects of *MMP‐9* knockdown. Consistent with these observations, artificial H3NT proteolysis at responsive gene promoters using unbiased CRISPR‐dCAS9 targeting system emphasizes the functional significance of *MMP‐9* protease activity toward H3NT in driving oncogenic gene transcription. These results unveil a previously uncharacterized function of nuclear *MMP‐9* and underscore the diagnostic, prognostic, and therapeutic potential of H3NT clipping to prevent the onset of colonic tumorigenesis.

## Author contributions

YS, and WA conceived and designed the study. YS performed experiments with contributions of WA. YS, SK, GL, and WA analyzed data. YS and WA wrote the manuscript. All authors read and approved the final manuscript.

## Conflict of interest

The authors declare no conflict of interest.

## Supporting information


**Fig. S1.** High levels of MMP‐9 expression in colon cancer.
**Fig. S2.** Cellular localization of MMP‐9 in colon cancer cells.
**Fig. S3.** Stable association between MMP‐9 and H3 in colon cancer cells.
**Fig. S4.** Heatmap representation of MMP‐9‐responsive genes.
**Fig. S5.** dCas9‐MMP‐9‐driven activation of target genes.
**Fig. S6.** dCas9‐MMP‐9‐driven H3NT proteolysis at target genes.
**Fig. S7.** dCas9‐MMP‐9‐driven enhancement of cell growth.
**Fig. S8.** H3NT proteolysis‐dependent function of MMP‐9 in colon cancer.

**Table S1.** List of the primers used in RT‐qPCR.
**Table S2.** List of the primers used in ChIP‐qPCR.
**Table S3.** Sequences of sgRNAs.

## Data Availability

Supporting data are available as supplementary Information. The gene expression array data has been deposited in the NCBI Gene Expression Omnibus (GEO) database under the GEO accession number GSE242924.
