# Supplementary material for: MMP‐9‐dependent proteolysis of the histone H3 N‐terminal tail: a critical epigenetic step in driving oncogenic transcription and colon tumorigenesis
Source: Mol Oncol. 2024 Apr 10;18(8):2001–19. doi: 10.1002/1878-0261.13652 (PMC11306514; doi:10.1002/1878-0261.13652)

Supplementary Fig. S1

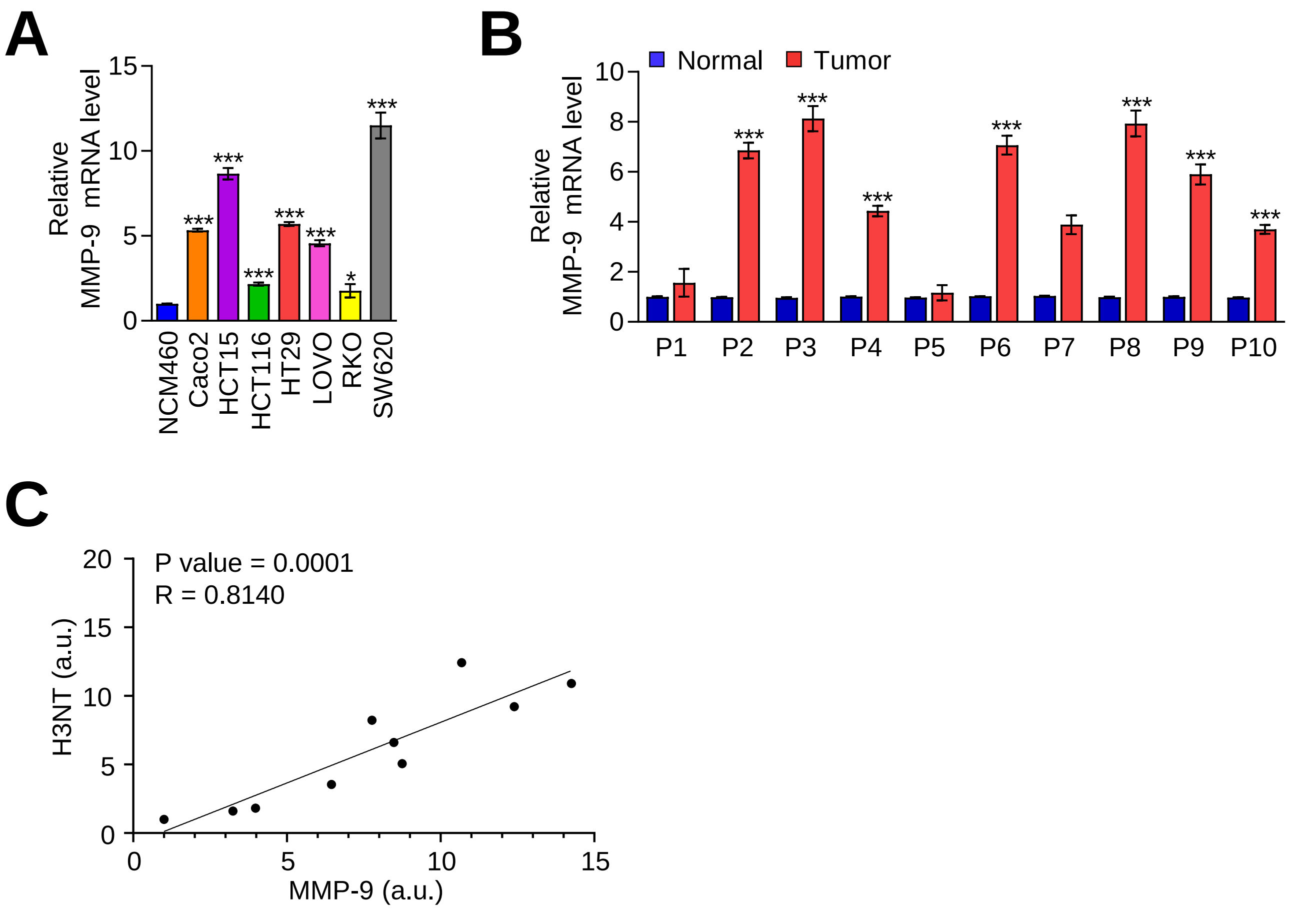

Supplementary Fig. S2

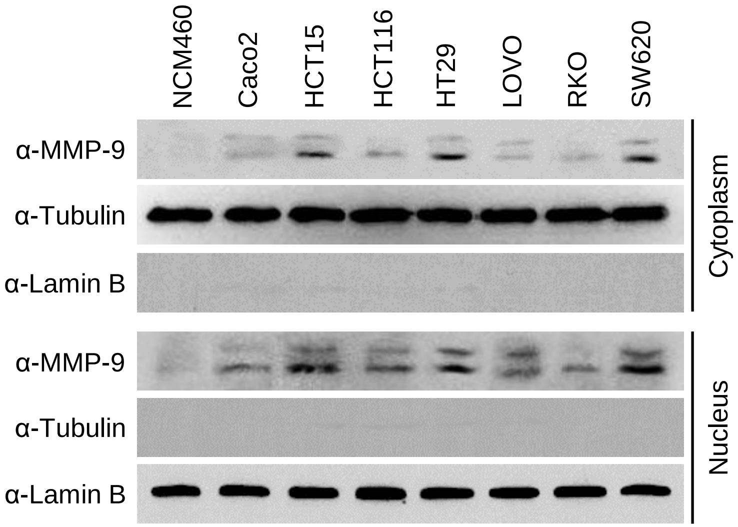

Supplementary Fig. S3

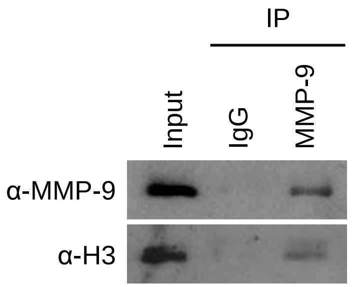

Supplementary Fig. S4

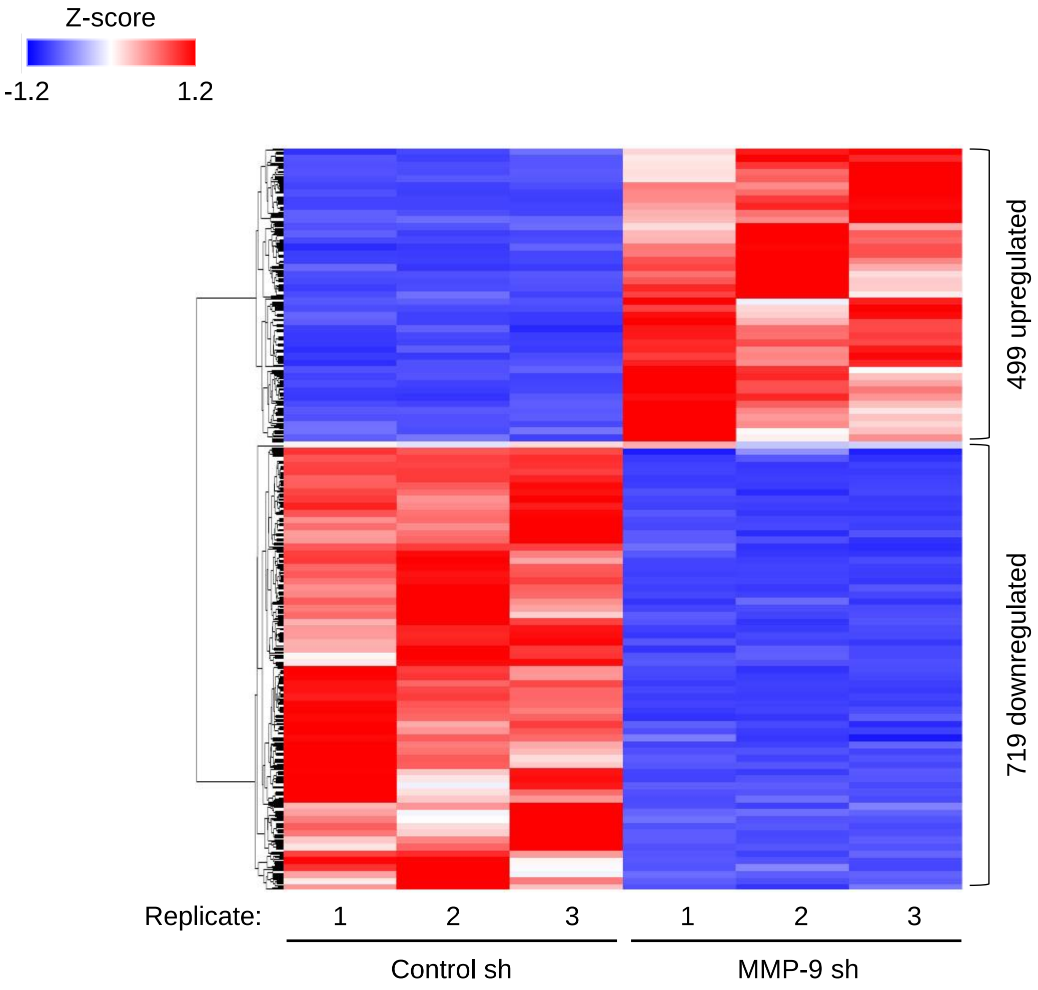

Supplementary Fig. S5

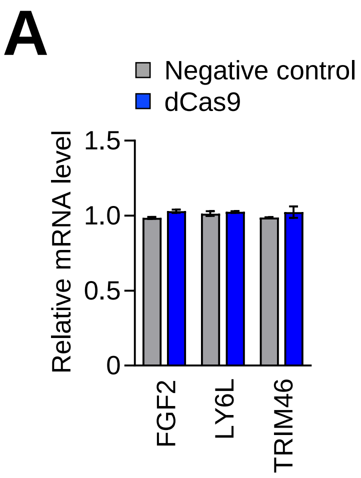

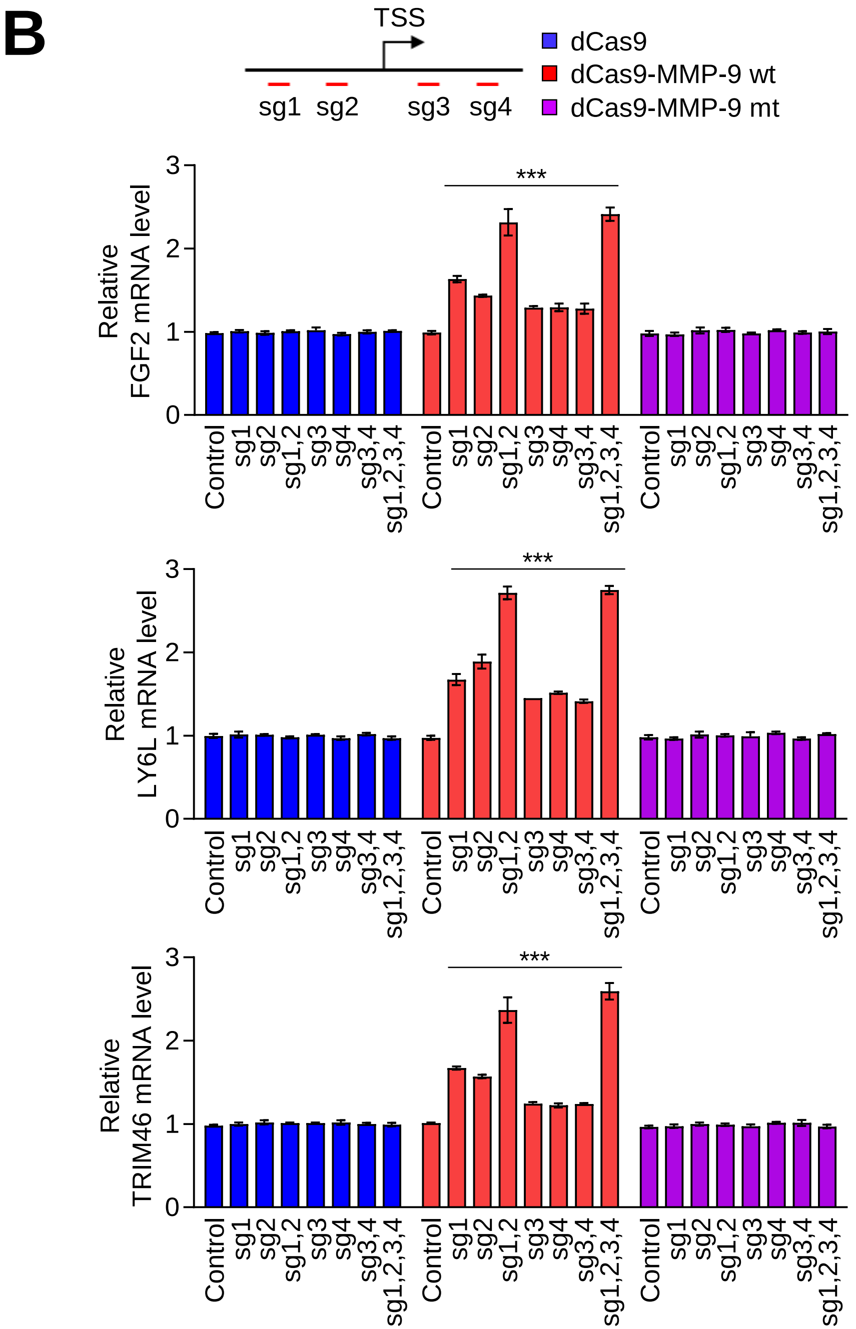

Supplementary Fig. S6

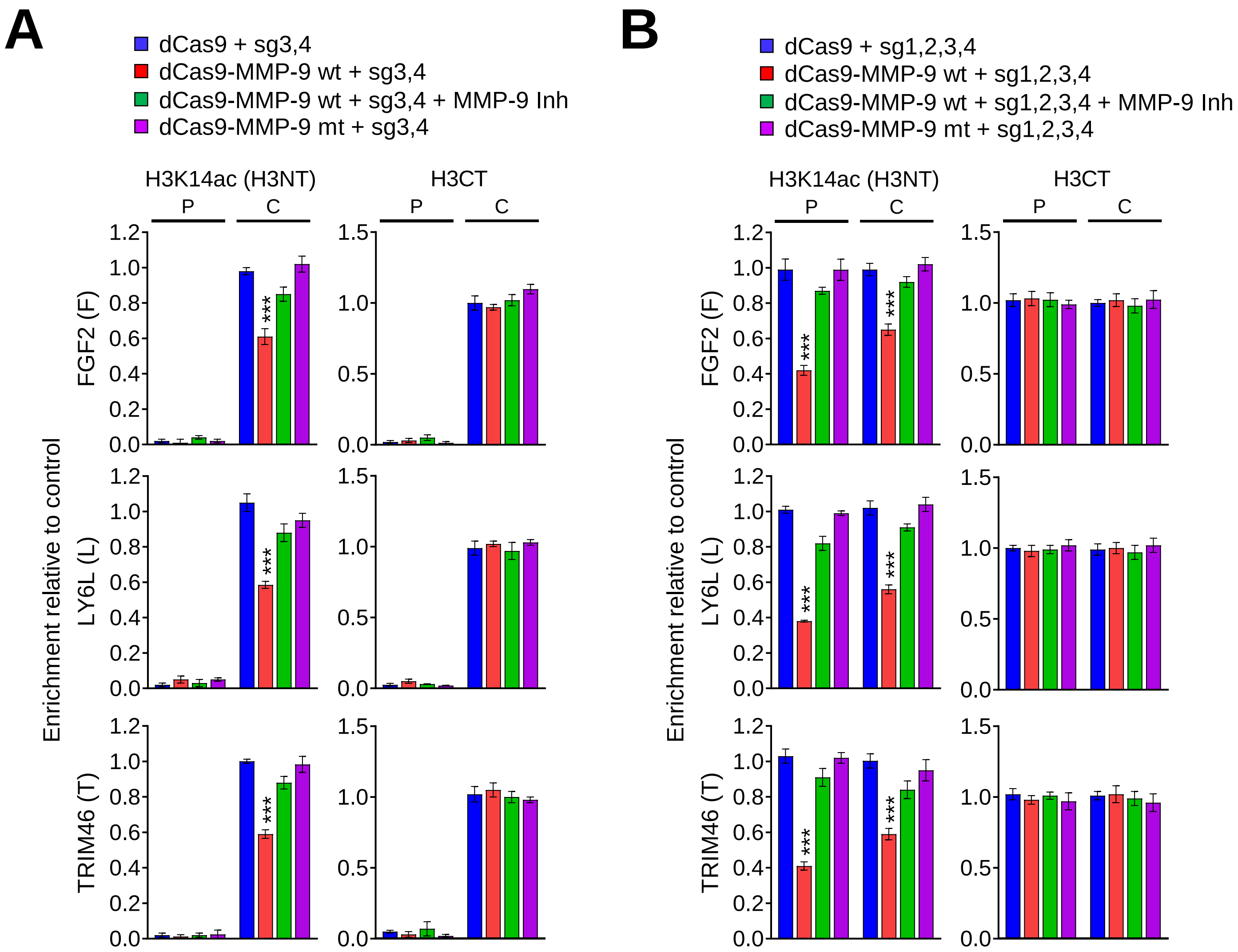

Supplementary Fig. S7

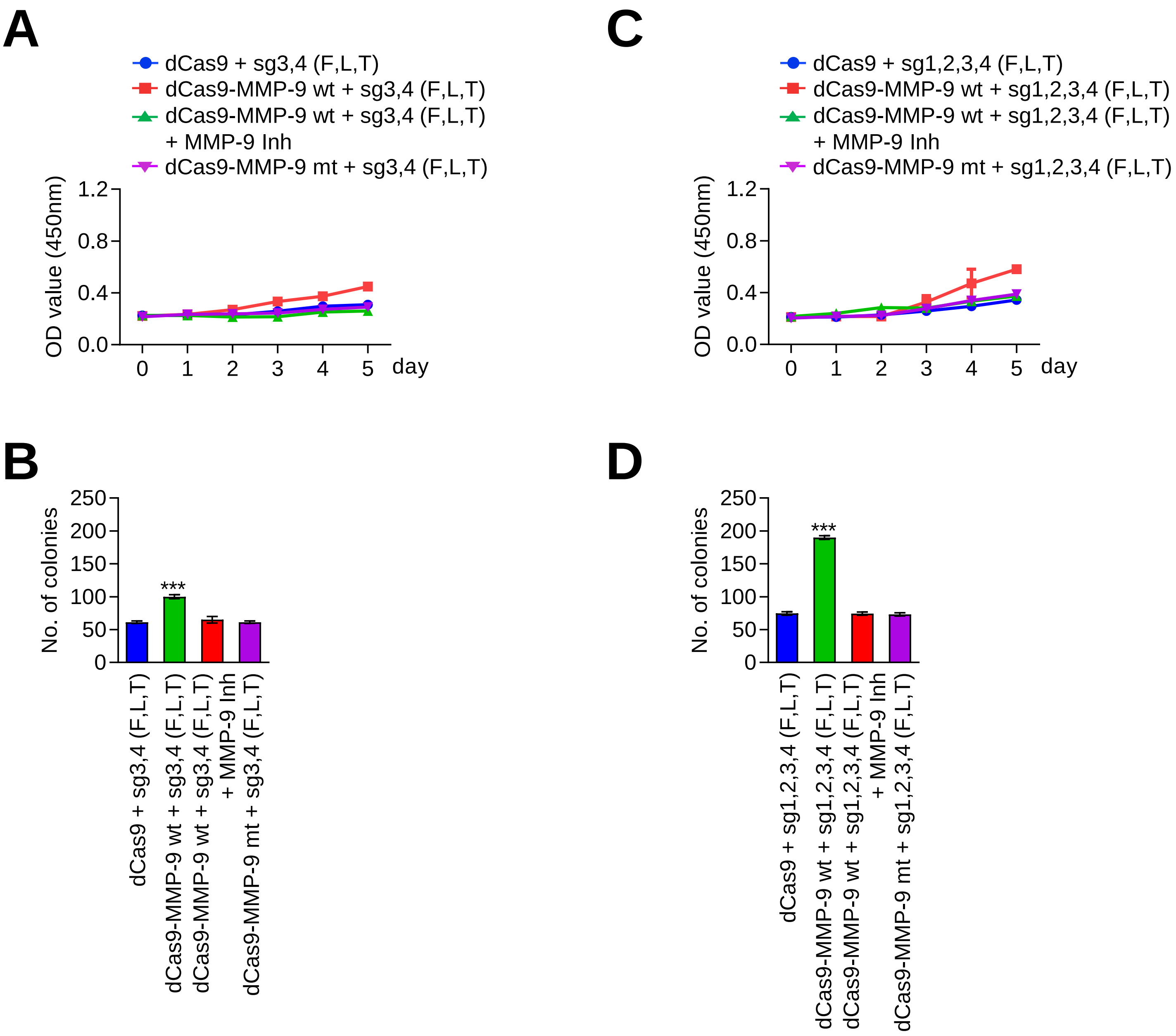

Supplementary Fig. S8

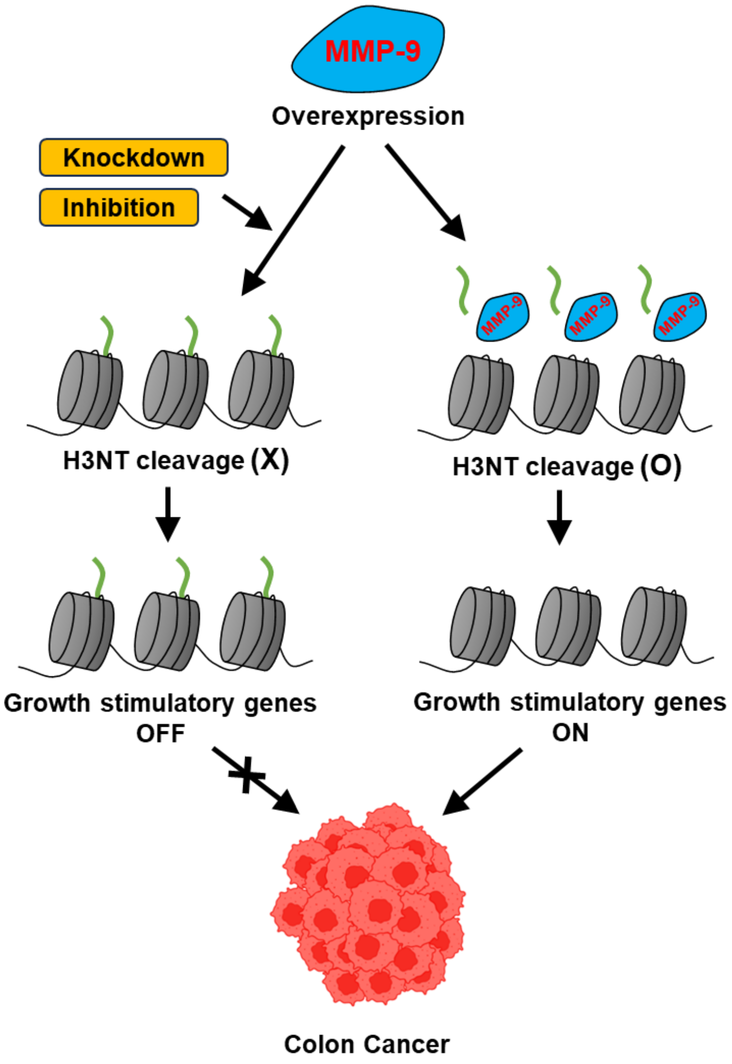

Supplement: Supplementary file 1 — Fig. S1. High levels of MMP‐9 expression in colon cancer. Fig. S2. Cellular localization of MMP‐9 in colon cancer cells. Fig. S3. Stable association between MMP‐9 and H3 in colon cancer cells. Fig. S4. Heatmap representation of MMP‐9‐responsive genes. Fig. S5. dCas9‐MMP‐9‐driven activation of target genes. Fig. S6. dCas9‐MMP‐9‐driven H3NT proteolysis at target genes. Fig. S7. dCas9‐MMP‐9‐driven enhancement of cell growth. Fig. S8. H3NT proteolysis‐dependent function of MMP‐9 in colon cancer. [file MOL2-18-2001-s001.pdf]
