# Supplementary material for: MMP‐9‐dependent proteolysis of the histone H3 N‐terminal tail: a critical epigenetic step in driving oncogenic transcription and colon tumorigenesis
Source: Mol Oncol. 2024 Apr 10;18(8):2001–19. doi: 10.1002/1878-0261.13652 (PMC11306514; doi:10.1002/1878-0261.13652)
Supplement: Supplementary file 2 — Table S1. List of the primers used in RT‐qPCR. Table S2. List of the primers used in ChIP‐qPCR. Table S3. Sequences of sgRNAs. [file MOL2-18-2001-s002.docx]

**Supplementary figure legends**

**Fig. S1. High levels of MMP-9 expression in colon cancer.**

(A) Total RNA was isolated from colon cancer and normal colon cells used in Fig. 1A and subjected to RT-qPCR with MMP-9 specific primer set listed in Supplementary Table S1. Data are represented as the mean ± standard deviation (SD) from triplicate RT-qPCR analysis; **P* < 0.05, ****P* < 0.001 compared to control sh.

(B) RT-qPCR was conducted to quantify MMP-9 mRNA levels in ten human colon tumors and their adjacent normal tissue samples. Data are represented as the mean ± SD from triplicate RT-qPCR analysis; ****P* < 0.001 compared to normal.

(C) Pearson’s correlation coefficients (r) were calculated between MMP-9 and H3NT proteolysis shown in Fig. 1A, and a dot of scatter plot represents the individual score of each of protein abundance. *P* value is determined by two-sided Pearson’s correlation test using GraphPad Prism 9 software.

**Fig. S2.** **Cellular localization of MMP-9 in colon cancer cells.**

Cytoplasmic and nuclear extracts were prepared from NCM460, Caco2, HCT15, HCT116, HT29, LOVO, RKO, and SW620 cells and analyzed by Western blotting with MMP-9 antibody. Lamin B and Tubulin are nuclear and cytoplasmic markers and served as loading controls.

**Fig. S3. Stable association between MMP-9 and H3 in colon cancer cells.**

Endogenous MMP-9 was immunoprecipitated from cell lysates prepared from SW620 cells. The presence of H3 in the precipitate was confirmed by Western blot analysis.

**Fig. S4. Heatmap representation of MMP-9-responsive genes.**

A heatmap shows 499 upregulated genes and 719 downregulated genes in response to MMP-9 knockdown in SW620 cells.

**Fig. S5. dCas9-MMP-9-driven activation of target genes.**

(A) SW620 cells were transfected with negative control and dCAS9 only constructs, and

total RNA was isolated and analyzed by RT-qPCR using primers specific for the FGF2, LY6L, and TRIM46 genes. Data represents as the mean ± S.D obtained from independent triple experiments.

(B) SW620 cells were transfected with sgRNA and dCas9 expression constructs (dCas9, dCas9-MMP9 wt, or dCas9-MMP-9 mt). RT-qPCR experiments were performed using primers specific for FGF2, LY6L and TRIM46 genes as in Fig. 6. Data represents the mean ± SD obtained from independent triple experiments; ****P* < 0.001 versus control.

**Fig. S6. dCas9-MMP-9-driven H3NT proteolysis at target genes.**

(A) MMP-9-depleted SW620 cells were transfected with dCas9-MMP-9 and sgRNA 3 and 4 pair expression constructs targeting coding regions of FGF2, LY6L and TRIM46 genes in the absence or presence of the MMP-9 inhibitor MMM9-I. The levels of H3K14ac at the promoters and coding regions of the genes were assessed by ChIP-qPCR. ***p < 0.001.

(B) ChIP assays were performed as in A but using cells transfected with sgRNA 1, 2, 3 and 4 together. ****P* < 0.001.

**Fig. S7. dCas9-MMP-9-driven enhancement of cell growth.**

(A) dCas9-MMP-9 was guided to the coding regions of target genes as in supplementary Fig. 5A, and changes in cell growth were monitored by MTT assays over a period of 5 days. The results represent the mean ± SD of three experiments performed in triplicate.

(B) Colony formation assays were performed with SW620 cells after selective upregulation of FGF2, LY6L and TRIM46 genes using CRISPR/dCas9 system as in A. Data represents the mean ± SD of three independent experiments in triplicate wells; ****P* < 0.001.

(C) MTT assays were performed as in A but using cells transfected with sgRNA 1, 2, 3 and 4 together. The results are shown as the mean ± SD of three experiments conducted in triplicate.

(D) Colony formation assays were performed as in (B) but using cells transfected with sgRNA 1, 2, 3 and 4 together. Data represents the mean ± SD of three independent experiments, each performed in triplicate wells; ****P* < 0.001.

**Fig. S8.** **H3NT proteolysis-dependent function of** **MMP-9 in colon cancer.** A working model depicting the deregulated gene expression via MMP-9-dependent H3NT proteolysis during colonic tumorigenesis and the use of inhibition/knockdown approaches to modulate aberrant MMP-9 activity.

**Supplementary Tables**

**Table S1.** List of the primers used in RT-qPCR.

| Primers | Forward (5'-3') | Reverse (5'-3') |
| --- | --- | --- |
| FGF2 | aggagtgtgtgctaaccgtt | cagttcgtttcagtgccaca |
| LY6L | ctcgtcctaaccctgtgca | cgttggagatgcagacttgg |
| MAPK4 | aagtacatccactccgccaa | ccaacccgaaatccccaatc |
| NMNAT2 | gtggagcgtttcacctttgt | cacctccatatctgcctcgt |
| OSBPL6 | gccattcaacccagtccttg | aggcagaaatgggtggatga |
| SAA1 | gaagtgatcagcgatgccag | cagcaggtcggaagtgattg |
| SYT11 | aagccgagacaaagatccca | agcatgacatcctcctctgg |
| TRIM46 | acccttcgctttcctaacca | ccacggaaggaaacagcatc |
| MMP-9 | gacaagaagtggggcttctg | ccctcagtgaagcggtacat |
| GAPDH | AACTTTGGCATTGTGGAAGG | GGATGCAGGGATGATGTTCT |

**Table S2.** List of the primers used in ChIP-qPCR.

| Primers | Forward (5'-3') | Reverse (5'-3') |
| --- | --- | --- |
| FGF2 (P) | ccctctcccatcccattacc | gcttgaaggagatgaaggcg |
| FGF2 (C) | gtgtgctaaccgttacctgg | ctgcccagttcgtttcagtg |
| LY6L (P) | cagaggacaaagggagcaga | atttcccctgcgtccacata |
| LY6L (C) | ccaagtctgcatctccaacg | gatcacgccttgcaccatg |
| TRIM46 (P) | tgggaggggattaggaggaa | tcatccctttccctgaccac |
| TRIM46 (C) | tgcccagtgtgtcaagagat | gtccccaccatgtcctatgt |

**Table S3.** Sequences of sgRNAs.

| Genes | Sequences (5’- 3’) |
| --- | --- |
| FGF2 (sg1) | GGTGAACCCGAAACCGCCGA |
| FGF2 (sg2) | GCGGACAACCTGTCGCGTCG |
| FGF2 (sg3) | CCCCGACGGCCGAGTTGACG |
| FGF2 (sg4) | CAACTTCAAGCAGAAGAGAG |
| LY6L (sg1) | GGCCGCGCCTTACCTGAGTG |
| LY6L (sg2) | GCACGTAACCTGGCCCTGCG |
| LY6L (sg3) | ACGACAACATGAAGTTCGAA |
| LY6L (sg4) | TGATCACGCCTTGCACCATG |
| TRIM46 (sg1) | GAATGCGTGACATCCTCGGT |
| TRIM46 (sg2) | ACCGGCTTTCGAGAACCACG |
| TRIM46 (sg3) | CATACACAGAGCCCGTGTCG |
| TRIM46 (sg4) | CGTGTCGGGGTTCTCAAGCA |
